# Traditional Chinese Medicine-Associated Bioactive Hydrogels: Material Roles, Network Design, and Therapeutic Applications

**DOI:** 10.3390/gels12100937

**Published:** 2026-10-20

**Authors:** Zile Song, Jiafei Chen, Chun Pu, Gaojie Xin, Qi Shi, Sijing Hu, Quanlong Zhang, Qiaoyan Zhang, Luping Qin, Min Hu

**Affiliations:** College of Pharmaceutical Science, Zhejiang Chinese Medical University, Hangzhou 310053, China

**Keywords:** traditional Chinese medicine, bioactive hydrogels, drug-as-material, disease-matched design, translational biomaterials

## Abstract

Traditional Chinese medicine (TCM)-associated hydrogels are structurally diverse systems in which medicinal constituents can function primarily as gelators, cooperating network components, matrix-functionalizing moieties, scaffold-forming medicinal polysaccharides, or therapeutic payloads. This diversity expands formulation possibilities but can obscure the respective contributions of material design, delivery, and pharmacological activity. This review proposes a role-based framework that distinguishes constituent identity and chemical complexity from experimentally demonstrated network function. It applies this framework to active-molecule self-assembly, multicomponent and metal-assisted assembly, polymer-supported integration, medicinal-polysaccharide networks, and gel formulations containing complex herbal preparations. Therapeutic applications are considered in relation to four recurring pathological and delivery constraints, including limited residence at dynamic interfaces, oxidative and inflammatory microenvironments, structurally deficient regenerative niches, and the need for spatial confinement when systemic or off-target exposure constrains treatments. Across these applications, this review assesses evidence for hydrated-network formation, component-resolved release and exposure, and appropriate comparators, while distinguishing structural cooperation from coordinated delivery and pharmacological synergy. Future progress will require reproducible feedstocks and processing, physiologically relevant characterization, manufacturing-aware design, and standardized datasets to support predictive modeling. This review provides a conceptual roadmap for the development and utilization of TCM-associated hydrogels as bioactive biomaterials.

## 1. Introduction

Traditional Chinese medicine (TCM) contains a wide range of chemically diverse bioactive constituents, including flavonoids, alkaloids, terpenoids, polyphenols, and polysaccharides, as well as complex multicomponent formulations. Many of these constituents possess anti-inflammatory, antioxidant, antimicrobial, immunomodulatory, or regenerative activities [1,2,3]. However, the therapeutic application of TCM-derived agents is often constrained by poor aqueous solubility, physicochemical instability, rapid systemic clearance, and uncontrolled tissue distribution [4,5]. In multicomponent formulations, these limitations are compounded by differences in the behavior of individual constituents. Differences in solubility, stability, pharmacokinetics, and tissue affinity may affect the proportions of individual constituents that ultimately reach the target site. The composition delivered to the target tissue may differ substantially from that of the original preparation, potentially weakening the coordinated multi-target effects on which many TCM formulations are based [6,7].

Hydrogels offer a promising material platform for addressing several of these limitations. Their highly hydrated networks, tunable crosslinking density, and tissue-like mechanical properties can be engineered to support local retention, controlled release, tissue adhesion, mechanical support, and microenvironmental regulation [8,9,10]. These properties are particularly valuable when therapeutic efficacy depends on maintaining local concentrations, preserving constituent stability, or adapting therapeutic exposure to changes in the pathological microenvironment. However, TCM-associated hydrogel systems differ in their underlying design principles. In many studies, a preformed gel functions primarily as a carrier for an extract, a purified compound, or a polysaccharide. Although this approach may improve local retention, the matrix remains functionally separate from the payload. A more distinctive class has recently attracted increasing attention, in which TCM-derived constituents participate directly in network formation or influence the properties of the material through mechanisms such as supramolecular self-assembly, dynamic covalent bonding, metal–phenolic coordination, polysaccharide crosslinking, or interfacial regulation [11,12,13,14]. In these systems, the active constituent is not simply incorporated as a therapeutic payload. It can also contribute to network stability, adhesion, or responsiveness to the disease environment.

This distinction motivates a shift from formulation-centered delivery to materials-centered design. The central question is not merely whether a hydrogel can carry an herbal constituent more efficiently, but whether that constituent can participate in constructing a material whose structure and therapeutic function are closely linked. From this perspective, progress should be evaluated not only by loading capacity, burst-release suppression, or short-term efficacy, but by the extent to which the material architecture contributes mechanistically to therapeutic effect. TCM concepts such as systems-level regulation, dynamic rebalancing, hierarchical cooperation, and site-directed therapeutic action may offer design inspiration rather than serve as direct molecular equivalents. Their usefulness lies in framing testable predictions concerning multiscale networks, responsive behavior, combinatorial bioactivity, and localized intervention.

**Figure 1:**
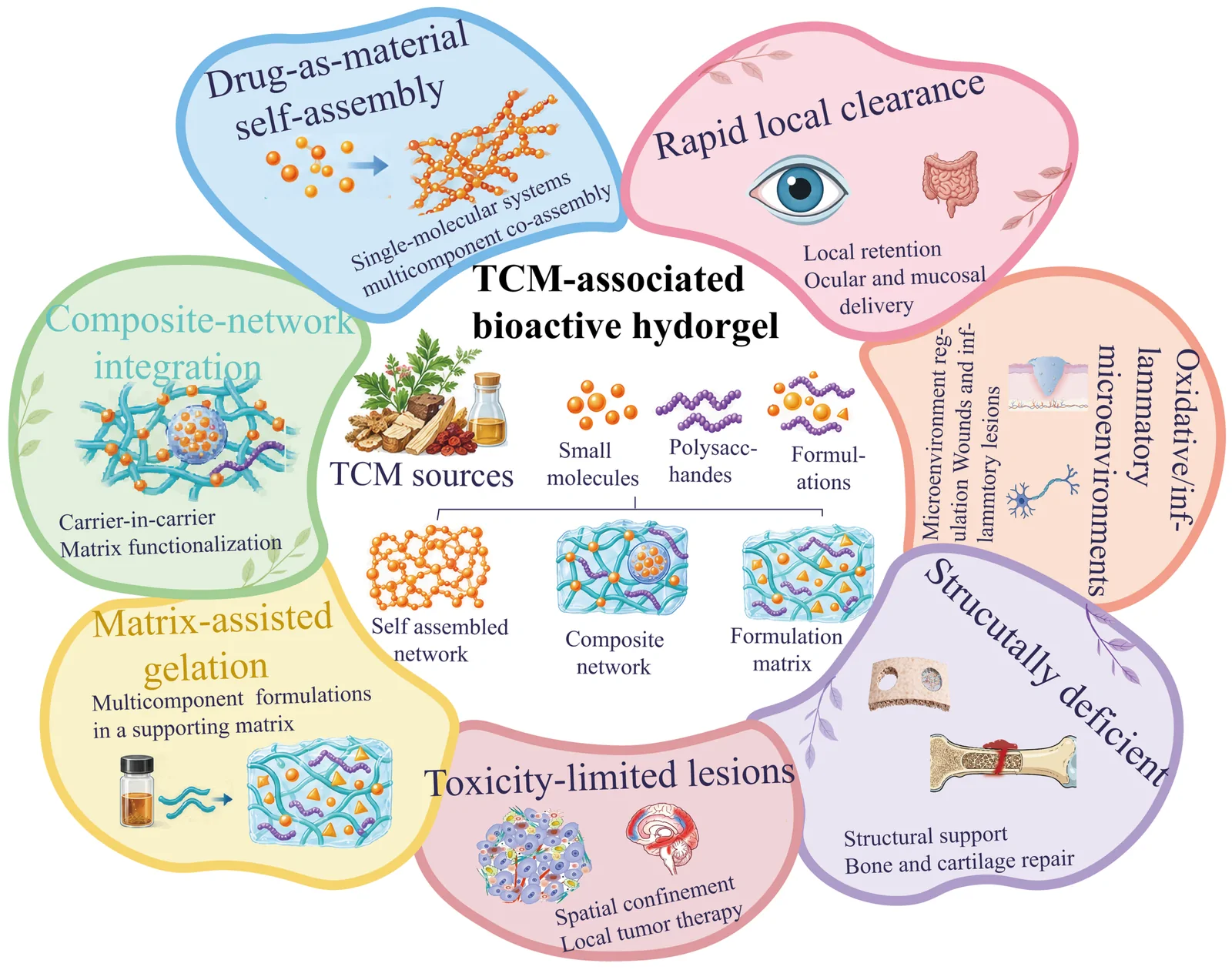
TCM-associated bioactive hydrogels and their applications.

In this review, we treat TCM-associated hydrogels as an emerging class of bioactive materials and organize the field according to the contributions of TCM constituents to network formation and function. We first translate selected elements of TCM therapeutic logic into testable materials hypotheses. We then distinguish three engineering routes: drug-as-material self-assembly, composite-network integration, and matrix-assisted gelation of multicomponent formulations. Next, we match these architectures to four pathological barriers, including rapid local clearance, oxidative and inflammatory stress, structurally deficient regenerative niches, and the need to confine toxicity-limited agents (Figure 1). Finally, we examine the challenges of predicting relationships between structure and assembly, establishing causality in multicomponent function, reproducibility, manufacturing, and regulatory positioning. The objective is to advance the field from empirical formulation towards predictive, mechanism-guided, and clinically relevant materials engineering.

### 1.1. Scope and Definitions

TCM-associated hydrogels have been widely reported. However, existing studies differ substantially in their conceptual frameworks, material complexity, and standards of evidence. Such differences make direct comparison challenging and make it difficult to determine how TCM-derived components contribute to hydrogel formation and function. A clear operational framework is therefore required to support a systematic evaluation of the existing literature.

Here, “TCM-derived” refers to molecules, fractions, or extracts obtained directly from TCM materials, such as glycyrrhizic acid from *Glycyrrhiza glabra*, rhein from *rhubarb*, and berberine from *Coptis chinensis*. “TCM-associated” refers to a hydrogel that contains at least one TCM-derived component that contributes to network formation, network function, or therapeutic activity within the hydrogel system. “TCM-inspired” is reserved for systems designed according to a TCM therapeutic principle that do not necessarily contain a TCM-derived component. These terms are distinct and are used consistently throughout this review.

Within the category of TCM-associated hydrogels, we distinguish three roles for TCM-derived components. In drug-as-material self-assembly, the TCM-derived component acts as a principal gelator or co-gelator, and its removal either prevents network formation or substantially alters the resulting network. In composite-network integration, an engineering polymer, nanoparticle assembly, inorganic phase, or hybrid scaffold forms the principal network, while the TCM-derived component makes a contribution to crosslinking, reinforcement, adhesion, antioxidant or antimicrobial activity, microenvironmental responsiveness, or other interfacial properties. In matrix-assisted gelation, a conventional gelator forms the principal network, and the TCM-derived preparation functions primarily as a therapeutic payload. This three-category framework, summarized in Box 1, is applied throughout Section 3 and Section 4.

Box 1 **Classification Criteria and Operational Definitions. Classificationis based first on the architecture of the hydrated network and then on the experimentally demonstrated role of the TCM-derived component. These categories describe distinct engineering routes rather than a ranking of scientific novelty or therapeutic efficacy.**

**Category**

**Operational Definition**

**Evidence Needed for Assignment**
Drug-as-material self-assemblyAssemblies formed primarily from one or more active small molecules constitute the continuous network, without a separate macromolecular scaffold dominating gel formation.Evidence of hydrated network formation, bulk viscoelastic behavior, and a relationship between molecular assembly and gelation.Composite-network integration: carrier-in-carrierA distinct drug-containing carrier phase is incorporated within a separate macroscopic gel network.Identification and characterization of both the embedded carrier phase and the surrounding hydrogel network, together with evidence of loading and spatial phase distribution.Composite-network integration: matrix functionalizationA scaffold is deliberately modified to bind, present, or respond to TCM-derived constituents, or incorporates those constituents as functional elements of the network.Evidence of chemical incorporation or defined molecular association, together with characterization of its effect on network, interfacial, or stimulus-responsive properties. Pendant functional groups should be distinguished from groups that contribute directly to network crosslinking.Matrix-assisted gelationA conventional gel-forming matrix primarily converts a therapeutic preparation into a retained dosage form, while the herbal constituents function mainly as payloads.Identification of the gel-forming matrix and formulation composition, supported by rheological characterization. Any advantage of gelation requires comparison with the corresponding nongelled preparation.

Boundary rules: (i) Molecular association is not sufficient evidence of gelation. Nanoparticles, micelles, precipitates, and organogels should not be classified as hydrogels unless the formation of a continuous hydrated network is demonstrated under the relevant experimental conditions. (ii) An active constituent is considered a payload when it is carried by the matrix without demonstrated participation in network formation or matrix functionalization. Antioxidant, antimicrobial, or other biological activity alone does not establish a structural role. (iii) Metal-dependent small-molecule networks are classified as multicomponent assemblies. The identity, chemical state, and amount of the metal component should be reported. “Carrier-free” does not mean metal-free, additive-free, or intrinsically safe. (iv) Host–guest interactions are classified according to their architectural role. A soluble inclusion complex incorporated into a conventional hydrogel does not constitute a co-gelator. By contrast, polymer-bound host–guest interactions that contribute to network connectivity, crosslinking, or defined molecular affinity are considered a form of matrix functionalization. (v) A medicinal polysaccharide that forms the scaffold is considered a network-forming polymer. Its incorporation into an engineered hybrid network falls under composite-network integration, whereas its simple dispersion within an independently formed matrix does not establish participation in scaffold formation. (vi) Hybrid formulations receive a primary classification according to their dominant macroscopic architecture, with secondary roles reported separately. (vii) Component-omission experiments can support role assignment but are not conclusive. Removing a constituent may simultaneously alter pH, ionic strength, solvent composition, total solids content, or other formulation variables. These changes should be controlled when interpreting loss of gelation. (viii) Structural cooperation, coordinated delivery, and pharmacological synergy represent distinct claims. They require evidence of altered network formation or material properties, component-resolved evidence of release or exposure, and evidence of a supra-additive biological effect evaluated using an appropriate additivity model.

This review focuses on systems in which TCM-derived components have a demonstrable role in hydrogel formation, function, or therapeutic activity. Systems in which a TCM component is simply dispersed in an unrelated hydrogel matrix without a measurable material or therapeutic advantage are excluded or discussed only as comparators. Converting an established herbal preparation into a hydrogel does not, by itself, constitute a substantive advance in materials design. The design must address a specific, measurable limitation of the original formulation, such as instability, inconsistent dosing, short residence time, inadequate local exposure, or limited local tolerability.

Classification is based on the architecture of the hydrated network and on the experimentally established role of the TCM-derived component, rather than on the mere presence of a particular interaction motif. For example, host–guest recognition may drive co-assembly, enable matrix functionalization, or primarily improve solubilization within a preformed matrix. The same interaction motif may therefore be involved in more than one engineering route.

### 1.2. Review Methodology

This review is a critical narrative review rather than a systematic review. We do not claim comprehensive coverage of all published work on TCM-associated hydrogels. Our aim is to organize and evaluate the literature on TCM-associated hydrogels according to the roles that TCM-derived constituents participate in hydrogel formation, network function, and therapeutic activity. The review draws on representative primary studies to illustrate the engineering routes, pathological barriers, and evidentiary standards discussed in Section 3, Section 4 and Section 5.

The search strategy was designed accordingly. We searched PubMed, Web of Science, and Scopus for articles published between January 2010 and June 2026. The main search strategy combined terms for TCM (“traditional Chinese medicine”, “TCM”, “herbal”, or “natural product”) with terms for hydrogels (“hydrogel”, “gel”, “supramolecular hydrogel”, or “*gel”) and therapeutic applications (“wound”, “inflammation”, ‘regeneration”, or “cancer”). Additional searches used the names of TCM-derived compounds frequently studied in hydrogel systems, including glycyrrhizic acid, rhein, berberine, curcumin, quercetin, baicalin, puerarin, and icariin. The reference lists of relevant reviews and primary studies were screened to identify additional eligible reports. The complete database-specific search strategies, including search strings, field tags, and number of records retrieved, are provided in Supplementary Methods S1.

After removing duplicates, studies were screened by title and abstract. Full texts were then assessed against the following criteria: (i) the study used a defined TCM-derived molecule, medicinal polysaccharide, extract, or named multi-herb preparation, (ii) the study reported a hydrated three-dimensional hydrogel network or an in situ hydrogel, and (iii) the study provided at least one material characterization or biological outcome. Studies were excluded if they investigated conventional hydrogels without a TCM-derived component, non-hydrated organogels that had not been converted into aqueous gels, or free TCM preparations without a hydrogel system. Studies were also excluded if they lacked sufficient formulation information to identify the network-forming component. Conference abstracts, patents, and non-peer-reviewed communications were excluded when they provided insufficient methodological detail.

Among the eligible pool, we selected studies for detailed discussion based on four considerations. First, we assessed their relevance to the role-based framework. Specifically, whether a TCM-derived component contributed to network formation, matrix functionalization, or therapeutic delivery, as distinct from simply being encapsulated. Second, we considered whether the study reported quantitative data on network formation, release behavior, or biological activity, as purely qualitative observations offer a limited basis for comparison. Third, we examined whether appropriate comparators were included, such as unassembled mixtures, polymer-only hydrogels, or free drug at equivalent dose. Finally, we sought to ensure representation across the three engineering routes and the four pathological barriers, avoiding an undue emphasis on any single design strategy or disease area.

The review has several limitations. The search was restricted to English-language records and three databases. The field is growing rapidly, and relevant studies may have been indexed after the search date or published in languages other than English. Many studies report gelation properties and in vitro activity, whereas fewer include in vivo validation, pharmacokinetic analyses, or safety assessments, and clinical outcomes are rarely reported. Rather than obscuring these limitations, we have sought to make them explicit and to indicate where claims extend beyond what the current evidence can support.

## 2. Translating TCM Therapeutic Logic into Testable Materials Hypotheses

**Figure 2:**
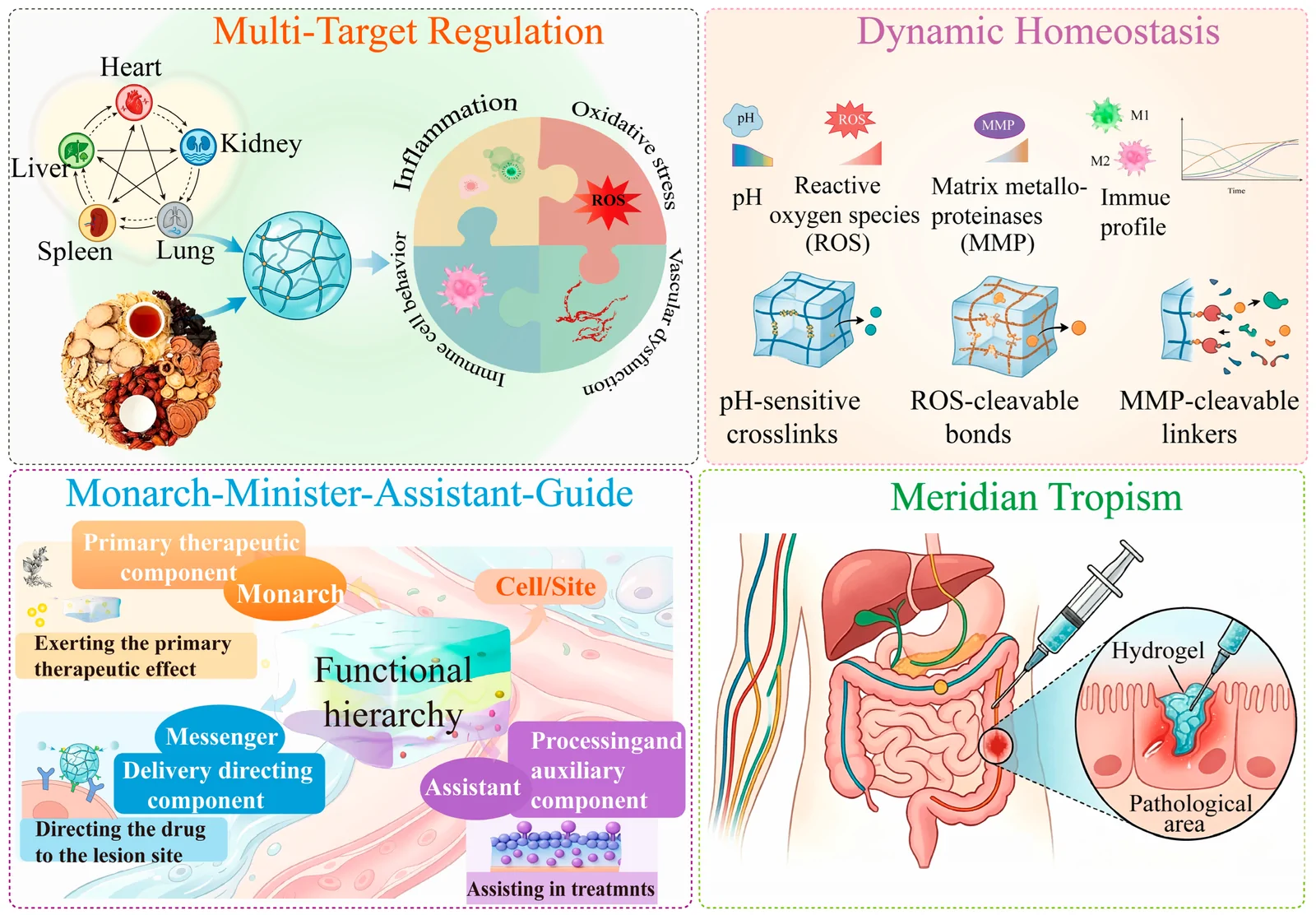
TCM theory-inspired design strategies for bioactive hydrogels.

Classical TCM theory and modern materials science rest on different assumptions about what constitutes knowledge and evidence. TCM concepts such as systems-level regulation, dynamic balance, and hierarchical cooperation were developed to describe patterns of illness and therapeutic responses at the level of the whole organism [15,16,17]. They were not formulated as molecular mechanisms and cannot be directly mapped onto defined biochemical pathways. Nevertheless, these concepts can suggest experimentally tractable materials hypotheses. For hydrogel design, the value of TCM therapeutic logic lies less in explaining molecular events than in posing testable questions about multicomponent interactions, responsive behavior, and localized intervention (Figure 2).

This perspective treats TCM as both a source of bioactive ingredients and a source of inspiration for materials design in complex disease microenvironments. Recent studies of programmable self-assembly of TCM-derived compounds have begun to establish a structure–assembly–property–function framework linking chemical features to supramolecular behavior and, ultimately, to therapeutic effects [18,19]. This framework provides a way to turn selected elements of TCM therapeutic logic into testable materials hypotheses without overstating the mechanistic equivalence between traditional concepts and modern molecular biology.

### 2.1. Systems-Level Regulation

Chronic wounds, neural injuries, and inflammatory mucosal diseases are not governed by a single molecule or pathway. They involve interconnected disturbances in inflammation, oxidative stress, vascular function, microbial balance, and immune-cell behavior [20,21]. A therapeutic strategy targeting only one of these processes may produce partial or transient benefits. TCM formulae have long combined multiple agents to address several aspects of a disease simultaneously.

Hydrogels can provide structural support, local retention, and spatiotemporally controlled presentation of bioactive cues within a single material platform. Their function therefore depends both on cargo release kinetics and on interactions at the material–tissue interface, which can influence several pathological processes at the same time. A recent review of TCM-based hydrogels for wound healing approaches this complexity through a hierarchy based on the number of components [22]. Single-component, dual-component, multicomponent, and formula-mimetic systems are presented as progressively more promising strategies for reproducing the broader regulatory effects associated with traditional formulae. This framework provides a basis for testing whether adding functional components leads to incremental improvements in therapeutic outcomes, whether benefits emerge only beyond a particular threshold, and whether the relationship between component number and efficacy differs across disease contexts.

### 2.2. Dynamic Rebalancing

Disease microenvironments are not static. During injury, inflammation, and tissue repair, local pH, reactive oxygen species (ROS) levels, enzyme activity, temperature, and cellular composition can change [23,24,25]. Hydrogels that respond to these signals by altering their swelling, degradation, crosslinking density, or release behavior may therefore adjust therapeutic output according to the local disease state. Such responsiveness provides a materials-level analogue to the TCM principle of adapting treatment to the stage, pattern and evolution of illness.

TCM staging is a clinical and phenomenological concept, whereas hydrogel responsiveness is a physicochemical property that can be characterized directly. The comparison is useful because it raises several experimentally testable questions about material design. For example, it remains unclear whether a hydrogel designed to respond to elevated ROS levels can achieve better therapeutic outcomes than a non-responsive hydrogel delivering the same total dose, or whether the optimal response threshold varies between acute and chronic inflammatory states. Recent studies of metal–phenolic networks formed from TCM-derived polyphenols and metal ions suggest that coordination bonds can provide dynamic crosslinking, while the resulting networks also exhibit ROS-scavenging activity, coupling network responsiveness to therapeutic function [26]. However, whether this coupling offers a therapeutic advantage over non-responsive systems with equivalent antioxidant capacity has yet to be systematically evaluated.

### 2.3. Functional Hierarchy: The Monarch–Minister–Assistant–Guide Principle

The monarch–minister–assistant–guide principle, known in Chinese as Jun-Chen-Zuo-Shi, describes the functional roles of ingredients in a TCM formula and informs their selection and relative proportions. Applied to biomaterials, this principle can be viewed as a hypothesis for establishing functional hierarchy in a multicomponent hydrogel. A “monarch” component may provide the primary therapeutic or structural function, while “minister” components may enhance bioactivity, mechanical stability, or interfacial properties. “Assistant” components may contribute to the regulation of degradation, responsiveness, or compatibility with the local microenvironment, while “guide” components could promote localization, tissue adhesion, or cell recruitment [27,28]. The purpose of this classification is not simply to assign traditional labels to individual ingredients, but to examine whether such a hierarchy can maintain the intended component ratios, support temporally ordered activity, or generate emergent functions that cannot be explained by the individual components.

### 2.4. Site-Directed Therapeutic Action

Meridian tropism provides a conceptual framework within TCM for describing the distribution of therapeutic effects. However, it should not be interpreted as equivalent to anatomical targeting, molecular recognition, or retention at a specific site of administration [29]. Its main relevance is to prompt questions about the spatial distribution of therapeutic activity, rather than to explain the mechanisms of tissue targeting. The mechanisms underlying hydrogel localization, component release, and subsequent tissue responses should therefore be established independently through appropriate experimental evidence.

Injectable, adhesive, and in situ-forming hydrogels may be designed to increase local residence and regulate the release of therapeutic components. A testable hypothesis is that these properties maintain effective exposure at the intended site while limiting unnecessary systemic exposure. Appropriate controls should include the corresponding free formulation, as well as a formulation in which the proposed retention or adhesion mechanism is selectively removed or modified. Such comparisons are important for determining whether improved therapeutic outcomes can be attributed specifically to enhanced local retention rather than to other differences between the formulations.

The residence time of the hydrogel matrix should be assessed separately from that of the active components it carries. A matrix may remain at the treatment site and continue releasing a compound even as that compound is rapidly cleared from the surrounding tissue, while a persistent imaging signal from the carrier does not necessarily establish the concentration or bioavailability of the active molecules. Delivery performance should therefore be assessed through measurements of release and local concentrations for individual components, alongside systemic exposure and relevant biological responses. Reduced systemic exposure and an improved therapeutic index should be demonstrated directly rather than inferred from local administration.

### 2.5. From Analogy to Testable Hypothesis

The four concepts discussed above are useful when they lead to specific and testable questions about materials design. They should not, however, be regarded as substitutes for experimental evidence of network formation, controlled delivery, biological activity, or safety. For each proposed conceptual relationship, the corresponding hypothesis should define the relevant variable, state the expected material or biological response, identify an appropriate comparator, and specify the measurements needed to evaluate the prediction.

This perspective has direct implications for the methodological design of materials research. Relationships among molecular structure, assembly pathways, network dynamics, release behavior, and pathological context should be examined through direct measurements and appropriately designed controls. A claim of synergy in a multicomponent hydrogel, for example, requires evidence that the combined effect exceeds the expected additive effect under controlled exposure conditions. Demonstrating that the combination performs better than an individual component is not sufficient to establish synergy. Similarly, adaptation to the disease microenvironment should be linked to a measurable therapeutic benefit. This demands comparison with an otherwise comparable non-adaptive system to establish whether the adaptive response improves the therapeutic outcome. When a material is described as mimicking a particular aspect of TCM therapeutic logic, the relevant conceptual elements should also be clearly defined and their individual contributions should be experimentally distinguishable.

Such an approach provides a basis for distinguishing substances in materials design from the simple incorporation of herbal constituents into a hydrogel matrix. It also provides a basis for the classification framework introduced in the following section, which organizes TCM-associated hydrogels according to the roles that TCM-derived constituents play in network formation and function. That framework, in turn, supports the assessment of the strength of evidence throughout this review.

## 3. Engineering Routes for TCM-Associated Hydrogels

TCM-derived constituents can be incorporated into a hydrogel as network-forming molecules, matrix-bound functional groups, encapsulated drugs, or components of a complex herbal preparation. These roles have different implications for material characterization and the interpretation of therapeutic effects. The presence of a bioactive constituent within a hydrogel does not establish its involvement in network formation. Likewise, evidence of molecular association or interaction is insufficient to establish the formation of a continuous hydrogel network.

Here, we organize the principal engineering strategies into three categories: drug-as-material self-assembly, composite-network integration, and matrix-assisted gelation of multicomponent formulations. Classification is based first on the architecture of the hydrated network and then on the experimentally supported role of each TCM-derived constituent. Drug-as-material systems are formed primarily through the assembly of active small molecules themselves. Composite-network systems incorporate a distinct carrier phase or deliberately functionalized scaffold, with the TCM-derived constituents contributing to the network or its functional properties. In matrix-assisted formulations, a gel-forming excipient primarily serves to convert a therapeutic preparation into a physically retained dosage form, without necessarily assigning the active constituents a structural role in network formation. Hybrid systems are classified according to their predominant network architecture, while secondary interactions and additional functional contributions are considered separately. The operational criteria used for this classification, together with representative boundary cases, are summarized in Box 1.

### 3.1. Drug-as-Material Self-Assembly

**Figure 3:**
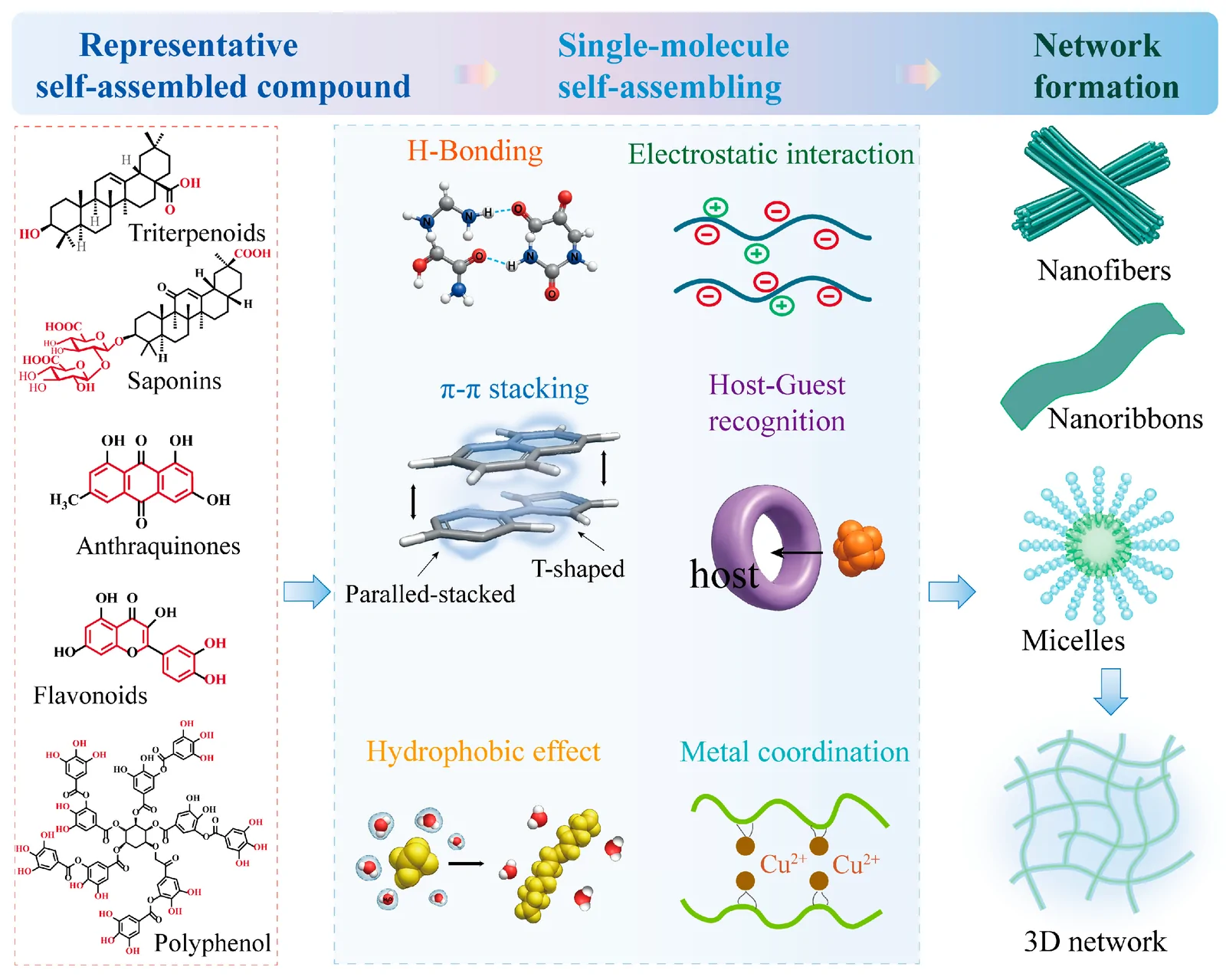
Drug-as-material self-assembly by TCM-derived small molecules. Cooperative non-covalent interactions that can drive supramolecular network formation.

Self-assembly most directly captures the drug-as-material concept in TCM-associated hydrogels. In this route, therapeutic molecules are not merely incorporated into a pre-existing matrix, but participate in the formation of the hydrogel network itself. Non-covalent interactions, including hydrogen bonding, π–π stacking, hydrophobic association, electrostatic attraction, host–guest recognition, and metal–ligand coordination, can drive TCM-derived molecules to assemble into nanofibers, ribbons, micelles, or other anisotropic aggregates that subsequently entangle, crosslink, or percolate into three-dimensional networks (Figure 3) [1,3,26,30,31]. The attraction of this strategy is its conceptual economy. The same molecule, or the same set of active molecules, may define both pharmacological function and network architecture.

The self-assembly of TCM-derived hydrogelators is rarely governed by a single molecular force. Instead, hydrogel formation usually emerges from a cooperative balance among aromatic stacking, hydrogen bonding, hydrophobic association, electrostatic interactions, coordination, and other weak interactions. The same interaction may promote gelation under one condition but lead to competing pathways, such as micellization, crystallization, or precipitation, under another. Molecular geometry, ionization state, solvent environment, concentration, ionic strength, and competition from biological species all influence whether a molecule forms a stable hydrogel network. Therefore, the central question is not simply whether a TCM-derived molecule contains aromatic rings, hydroxyl groups, ionizable groups, or amphiphilic domains, but whether these structural features can support a percolated, physiologically stable and therapeutically functional network. The major interactions involved in the self-assembly of TCM-derived hydrogelators are summarized in Table 1.

#### 3.1.1. Single-Molecule Self-Assembling Systems

Single-component self-assembly represents one of the most direct examples of the drug-as-material concept. In such systems, an individual TCM-derived active molecule spontaneously assembles into a supramolecular network through intermolecular interactions, thereby serving as both the therapeutic agent and the structural building block. Gelation may modify the apparent solubility, local retention, molecular presentation, and biological activity of the molecule. Although this strategy offers a high degree of conceptual simplicity, it imposes stringent demands on the molecular design. The molecule must combine sufficient bioactivity and dispersibility while providing enough intermolecular attraction to construct a stable network without undergoing precipitation or crystallization.

**Figure 4:**
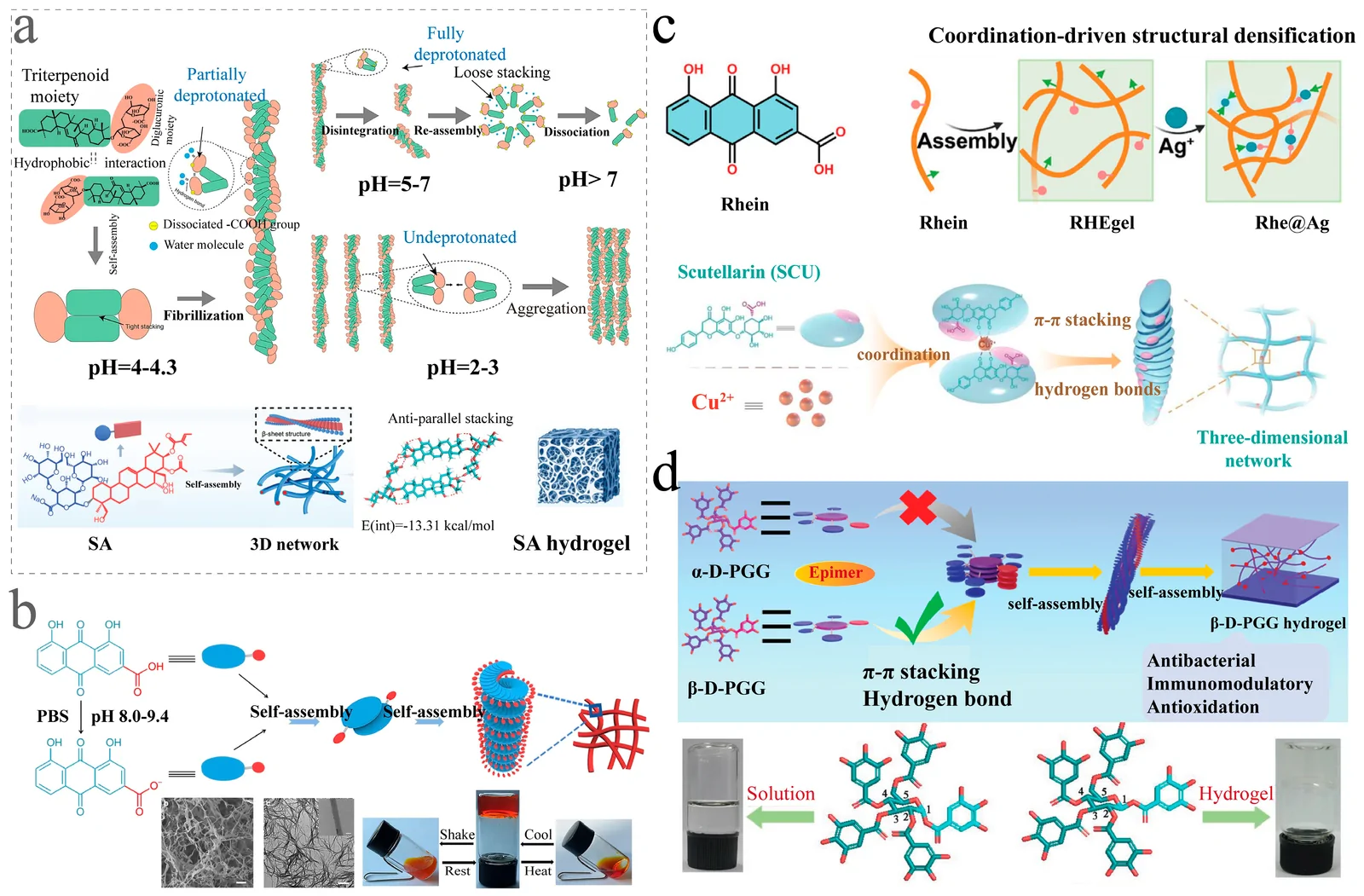
Drug-as-material self-assembly by TCM-derived small molecules. (**a**) pH-dependent assembly of glycyrrhizic acid into supramolecular hydrogel. Reproduced with permission from ref. [68]. Copyright 2025, Elsevier. (**b**) Formation of a rhein-based supramolecular hydrogel. Reproduced with permission from ref. [33]. Copyright 2025, Elsevier. (**c**) Representative metal-coordination hydrogels constructed from TCM-derived polyphenolic or related ligands and metal ions. Reproduced with permission from ref. [54]. Copyright 2024, John Wiley and Sons; reproduced with permission from ref. [79]. Copyright 2026, John Wiley and Sons. (**d**) Stereochemistry-dependent gelation of α-D-PGG and β-D-PGG. Reproduced with permission from ref. [44]. Copyright 2024, John Wiley and Sons.

Saponins and triterpenoids provide well-studied examples of this strategy. Glycyrrhizic acid (GA), a triterpenoid saponin derived from *Glycyrrhiza glabra*, is a representative system. Its hydrophobic triterpenoid backbone and hydrophilic sugar chains enable pH-dependent assembly [68]. Under acidic conditions, protonation of carboxyl groups favors hydrogen bonding and hydrophobic association, promoting the formation of left-handed helical nanofibers. At neutral pH, deprotonation increases electrostatic repulsion, which may favor the formation of nanoribbons or suppress gelation (Figure 4a). This pH-dependent polymorphism illustrates an important design consideration that amphiphilicity must be balanced to favor network percolation rather than micellization or precipitation. Related compounds, including zingibroside R1 [69], asiaticoside [41], sodium aescinate [49], oleanolic acid [45], betulinic acid [70], arjunolic acid [71], and ursolic acid [72], exhibit broadly similar assembly behavior, although their gelation windows and network stability differ according to molecular structure and environmental conditions.

Flavonoids and anthraquinones illustrate a complementary principle. Their rigid aromatic frameworks and multiple hydrogen-bonding sites can support supramolecular assembly, but only within a narrow energetic window that balances attractive interactions with sufficient dispersibility. Puerarin and quercetin have both been employed as building blocks for active supramolecular hydrogels [50,73,74]. Rhein, a major active component of *rhubarb*, is a representative TCM-derived hydrogelator capable of forming a gel through single-molecule assembly [33,75]. Its planar anthraquinone structure favors dimerization through strong face-to-face π-π stacking, while pH-dependent protonation of the carboxyl group can influence gel formation (Figure 4b). The assembly process is also affected by concentration and solvent polarity. Although the detailed assembly pathways may differ between individual compounds, successful gelation generally requires a balance in which intermolecular interactions are strong enough to support the formation of an ordered network without driving crystallization or precipitation. In practice, co-assembly with other molecules or incorporation into hybrid systems may produce a more robust route to gel formation than single-component formulations for some of these compounds.

Anthraquinone-based compounds also offer opportunities for the development of redox-responsive materials. Their quinoid structures can participate in electron donation and acceptance processes, suggesting the possibility of responding to redox-active pathological microenvironments. However, direct evidence that the intrinsic redox properties of pure anthraquinone compounds can be exploited to drive or regulate gelation remains limited. Further studies are therefore needed to establish whether redox processes directly alter material behavior and to distinguish such effects from the pharmacological effects of the unassembled compounds.

Polyphenols introduce additional functional dimensions to self-assembled TCM-derived hydrogels. In addition to contributing to supramolecular assembly [36,76], polyphenols can provide adhesive, antioxidant activity, and metal-binding capacity. Coordination with metal ions offers another route to network formation, although the resulting systems are multicomponent assemblies rather than strictly single-molecule systems. Metal–polyphenol networks formed by coordinating natural polyphenols, such as baicalin, tannic acid, gallic acid, rhein, or epigallocatechin-3-gallate, with metal ions like Zn^2+^, Fe^3+,^ Ce^4+^, Ag^+^ or Cu^2+^ have emerged as versatile hydrogel platforms [54,77,78,79] (Figure 4c). These networks can exhibit self-healing and pH-responsive behavior, together with ROS-scavenging or antimicrobial activity, thereby directly integrating TCM-derived molecules into the material framework. Nevertheless, polyphenolic systems are highly sensitive to molecular configuration and hydroxyl arrangement. For example, 1,2,3,4,6-penta-*O*-galloyl-β-*D*-glucose (β-*D*-PGG) can self-assemble into a gel under unmodified conditions, whereas its epimer, 1,2,3,4,6-penta-*O*-galloyl-α-*D*-glucose (α-*D*-PGG), does not readily assemble because its glycosidic configuration is less favorable for hydrogen bonding and π-π interactions [44] (Figure 4d). This comparison illustrates that assembly behavior cannot be predicted simply from the number of hydroxyl groups or aromatic rings present in a molecule. Instead, it depends on a combination of factors, including stereochemistry, packing geometry, solvation, and intermolecular coordination.

For single-molecule systems, the translational limitation is not simply whether a compound can form a gel in vitro, but whether the resulting network remains intact under physiologically relevant conditions. Gelation in purified buffer does not necessarily predict network stability in serum, wound exudate, mucus, synovial fluid, or other biological environments. Rigorous evaluation should therefore examine both rheological properties and assembly behavior in biologically relevant fluids, directly assess the integrity of the supramolecular network after administration, and distinguish therapeutic effects arising from molecular assembly from those attributable to the intrinsic pharmacological activity of the monomeric compound. A checklist for physiologically relevant characterization is provided in Supplementary Table S4.

#### 3.1.2. Multicomponent Co-Assembling Systems

In this review, multicomponent co-assembly is reserved for systems in which two or more molecular components, including at least one TCM-derived component, jointly form the continuous network. Host–guest recognition is discussed in this subsection because it is a common assembly motif, rather than a separate classification category. When host–guest recognition occurs within a functionalized polymer scaffold that provides the principal network, the system is classified as composite-network integration under Box 1, even though host–guest interactions also contribute to assembly or crosslinking. Such systems are treated here as boundary cases and are discussed further in Section 3.2.

Multicomponent co-assembly more closely reflects the multicomponent logic of many TCM formulations and the biological complexity of disease. The importance of these systems is not merely in incorporating otherwise non-gelling molecules into a hydrogel network, but in integrating pharmacological complementarity with structural cooperation and coordinated release within a single assembled material. Different components may nucleate assembly, reinforce the network, modulate solubility or responsiveness, and provide therapeutic activity.

**Figure 5:**
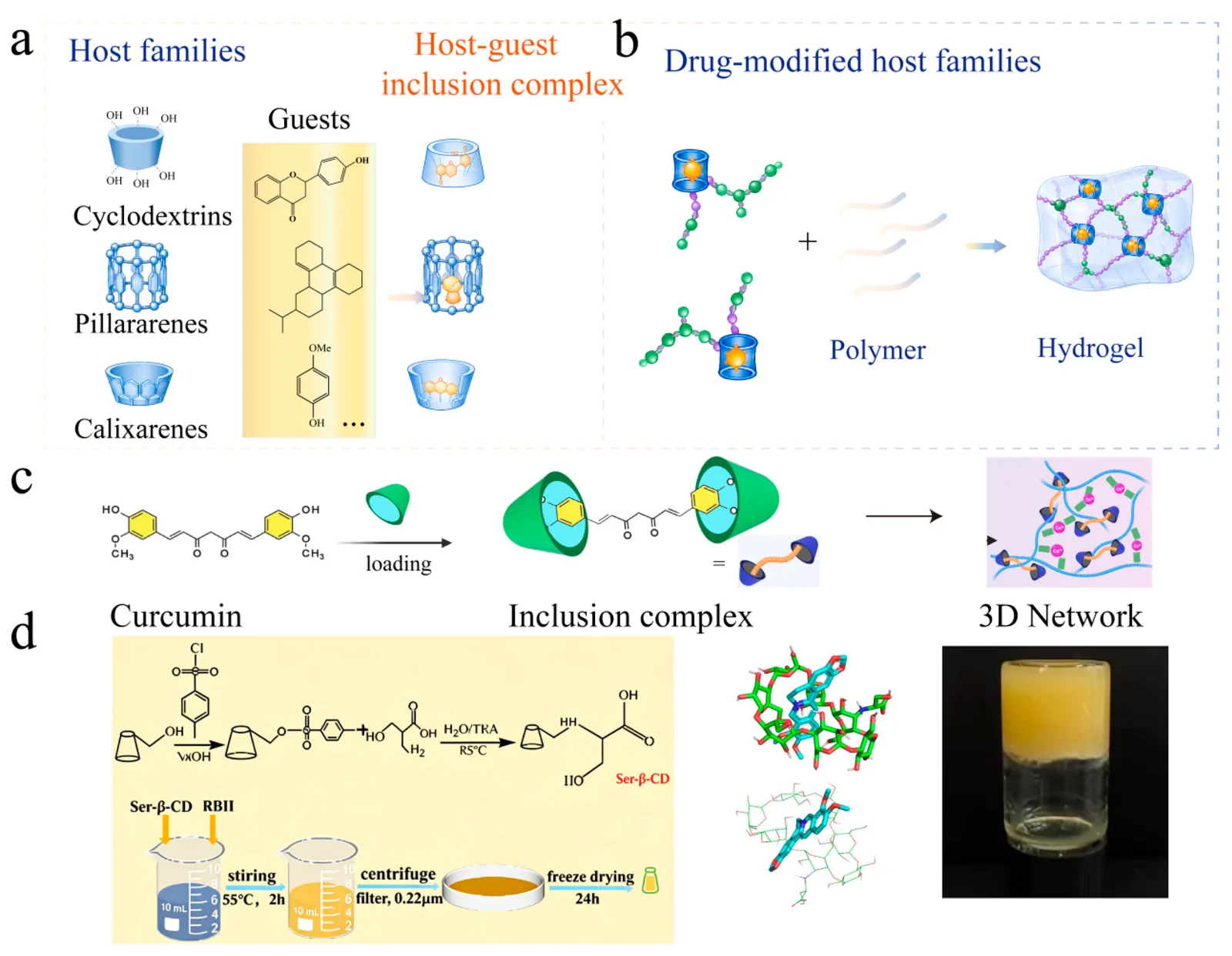
Host–guest and polymer-supported assembly systems. (**a**) Schematic illustration of hydrogel formation mediated by host–guest inclusion complexation. (**b**) Polymer-supported host–guest systems in which host motifs are associated with polymer backbones. (**c**) Formation of an alginate hydrogel based on host–guest inclusion complexation between (2-hydroxypropyl)-β-cyclodextrin (HPβCD) and curcumin. Reproduced with permission from ref. [61]. Copyright 2022, Elsevier. (**d**) Formation of a hydrogel using the berberine hydrochloride-ser-β-cyclodextrin inclusion complex. Reproduced with permission from ref. [82]. Copyright 2026, John Wiley and Sons.

Host–guest recognition offers one route to such cooperation. Macrocyclic hosts, including cyclodextrins, pillararenes, and calixarenes, can reversibly bind hydrophobic TCM-derived molecules or modified motifs, thereby improving solubility, stabilizing labile compounds and providing a basis for hydrogel formation (Figure 5a,c) [61,80,81]. When host motifs are grafted onto polymer backbones, such as hyaluronic acid, chitosan or polyethylene glycol, inclusion complexation can contribute to the formation of injectable supramolecular hydrogel networks (Figure 5b,d) [65,82]. Under the classification criteria in Box 1, systems in which a functionalized polymer scaffold forms the principal network belong to composite-network integration, even when host–guest recognition contributes to their assembly. The systems shown in Figure 5 are therefore presented as boundary cases rather than as strict examples of multicomponent co-assembly. Figure 5a,b involve polymer-supported host–guest architectures. When the functionalized polymer scaffold forms the continuous network, these systems are classified as composite-network integration. Figure 5c represents a related boundary case, in which an HPβCD–curcumin inclusion complex is incorporated into an alginate matrix [61]. If HPβCD remains free and functions only as a solubilizing agent, the system falls under matrix-assisted gelation. If HPβCD is covalently attached to the polymer and contributes to network connectivity, it falls under composite-network integration. The same criterion applies to Figure 5d. Classification is determined by the experimentally demonstrated role of each component in network formation, rather than the presence of host–guest recognition alone.

The principal value of host–guest systems is not only enhanced stability or solubility, but also their modularity and reversibility, which allow multiple components to be incorporated under relatively mild conditions. However, the introduction of host molecules also adds structural complexity. Complementary experimental evidence is therefore needed to distinguish network-forming supramolecular interactions from inclusion complexation that serves primarily to encapsulate a drug. Such evidence may include nuclear magnetic resonance spectroscopy, isothermal titration calorimetry, rheology, release studies and microstructural imaging.

Electrostatic co-assembly is another common strategy. Many TCM-derived active compounds contain ionizable groups, such as nitrogen-containing groups in alkaloids or carboxylates in phenolic acids and anthraquinones [83,84,85]. Attraction between oppositely charged species can drive ion pairing or polyelectrolyte complexation under mild aqueous conditions. The berberine-glycyrrhizic acid (BBR-GA) system is representative, as GA improves berberine solubility, while hydrogen bonding, aromatic interactions and electrostatic association collectively contribute to network stabilization [52]. However, in physiological saline environments, ionic competition may weaken the network, induce collapse or accelerate burst release. Moreover, complexes formed solely through electrostatic adsorption may be difficult to distinguish from simple co-deposition. Electrostatic interactions should therefore be considered alongside other potential cooperative driving forces, such as hydrogen bonding, π–π stacking or dynamic covalent bonds, rather than assumed to constitute the sole gelation mechanism.

A more ambitious form of co-assembly employs complementary components selected according to charge distribution, hydrophilic–hydrophobic balance, molecular geometry and aromatic packing compatibility. For example, a TCM-associated hydrogel composed of glycyrrhizic acid, berberine and rhein illustrates how a formula-inspired multicomponent network can be formed without relying on an inert carrier, with its constituents contributing both structural and therapeutic functions [86]. Such systems begin to approach the central ambition of TCM-associated hydrogel design, which is to organize multiple bioactive constituents into a material network in which assembly and therapy are mechanistically connected. By contrast, a system in which quercetin is incorporated into a zeolitic imidazolate framework (ZIF-8) and subsequently crosslinked with carboxymethyl chitosan is more appropriately considered a composite-network system [87], as discussed in Section 3.2.1.

Overall, multicomponent co-assembly represents an important step from proof-of-concept materials towards functionally integrated therapeutic platforms. However, a critical question remains unclear. Does the improved performance arise from emergent properties generated by co-assembly, or does it simply reflect localized co-delivery of multiple agents? Stronger claims require evidence of molecular-level co-assembly and a demonstration that release or local exposure differs from that of unassembled mixtures. The disease-relevant benefit should also be reduced or disappear when the assembled structure is disrupted; otherwise, it cannot be confidently ascribed to co-assembly.

#### 3.1.3. Critical Comparison

Single-molecule systems simplify compositional analysis and offer a relatively direct link between the active compound and network formation. Their main constraint is that drug dose, gelation and mechanics are coupled. Multicomponent assembly affords greater control over these properties, but introduces questions about which constituents drive assembly and how their composition changes during release. Meaningful comparisons must extend beyond the final gel modulus or the cumulative release of a single compound. Key parameters, including gelation concentration, pH, temperature, ionic strength, solvent content, and preparation history, should all be reported. Rheological comparisons should also be performed under comparable conditions, with measurements taken within the linear viscoelastic region where appropriate. Greater stiffness is not necessarily preferable. Materials intended for injection or mucosal contact have different mechanical requirements from scaffolds designed to maintain space within a tissue defect. Release measurements likewise require careful interpretation. For a poorly soluble compound, slow accumulation in the receiving medium may reflect limited dissolution rather than controlled release from the gel network. In multicomponent systems, the initial mixing ratio may differ from both the proportion incorporated into the network and the relative amounts released over time. Component-resolved measurements are therefore needed before coordinated exposure can be inferred. Gelation in a preparation buffer alone does not establish stability under physiological conditions. After administration, dilution, proteins, competing ions, and mechanical disturbance may disrupt or reorganize noncovalent networks. Characterization in relevant biological fluids should therefore assess mechanics, erosion, and component release together. The persistent fluorescent signal alone is not sufficient to establish that the original supramolecular structure remains intact. Biological studies should include the individual compounds and an unassembled mixture at justified doses. Where assembly-disrupting controls are used, changes in pH, solvent, or other chemical variables must be controlled so that they do not independently explain the biological response. Improved efficacy may arise from altered exposure without a new pharmacological interaction. Structural cooperation, coordinated delivery, and pharmacological synergy should therefore be evaluated as separate mechanistic claims.

### 3.2. Composite-Network Integration

Not all TCM-derived molecules are suitable for direct self-assembly, and a single-phase supramolecular network is often insufficient to meet the mechanical, pharmacokinetic and biological demands of complex therapeutic settings. Many herbal bioactives present difficulties because of high hydrophobicity, chemical lability, high dose demands, or the need to cross multiple biological barriers. Composite-network designs incorporate these constituents into a supportive polymer scaffold, either in their native form or after pre-organization into nanoscale domains. In such hybrid materials, the polymer network provides mechanical integrity, compliance suited to the tissue and spatiotemporal control of retention. The TCM-derived components supply therapeutic activity and may also contribute to network structure, interfacial properties, or responsiveness to the local environment.

#### 3.2.1. Hierarchical Carrier-in-Carrier Design

**Figure 6:**
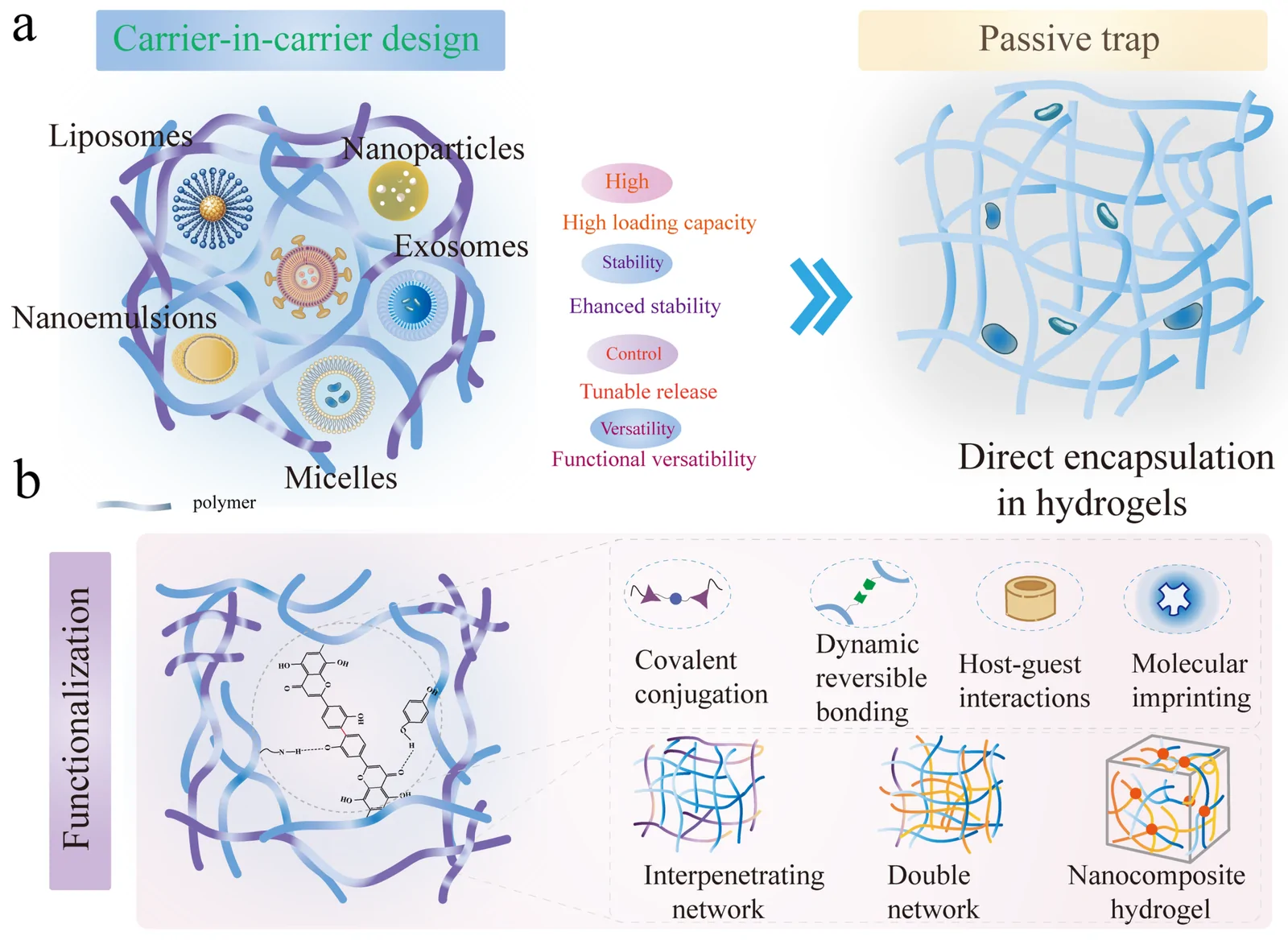
Schematic illustration of composite-network integrated strategies for TCM-derived hydrogels. (**a**) Hierarchical carrier-in-carrier design, in which TCM-derived bioactive components are first incorporated into nanoscale domains, such as liposomes, micelles, mesoporous particles, nanoemulsions or exosome-like vesicles, and then embedded within a macroscopic hydrogel matrix. (**b**) Matrix-functionalization strategy, in which TCM-derived bioactive components are grafted onto, coordinated with or otherwise integrated into polymer chains before polymerization, crosslinking or network formation.

**Figure 7:**
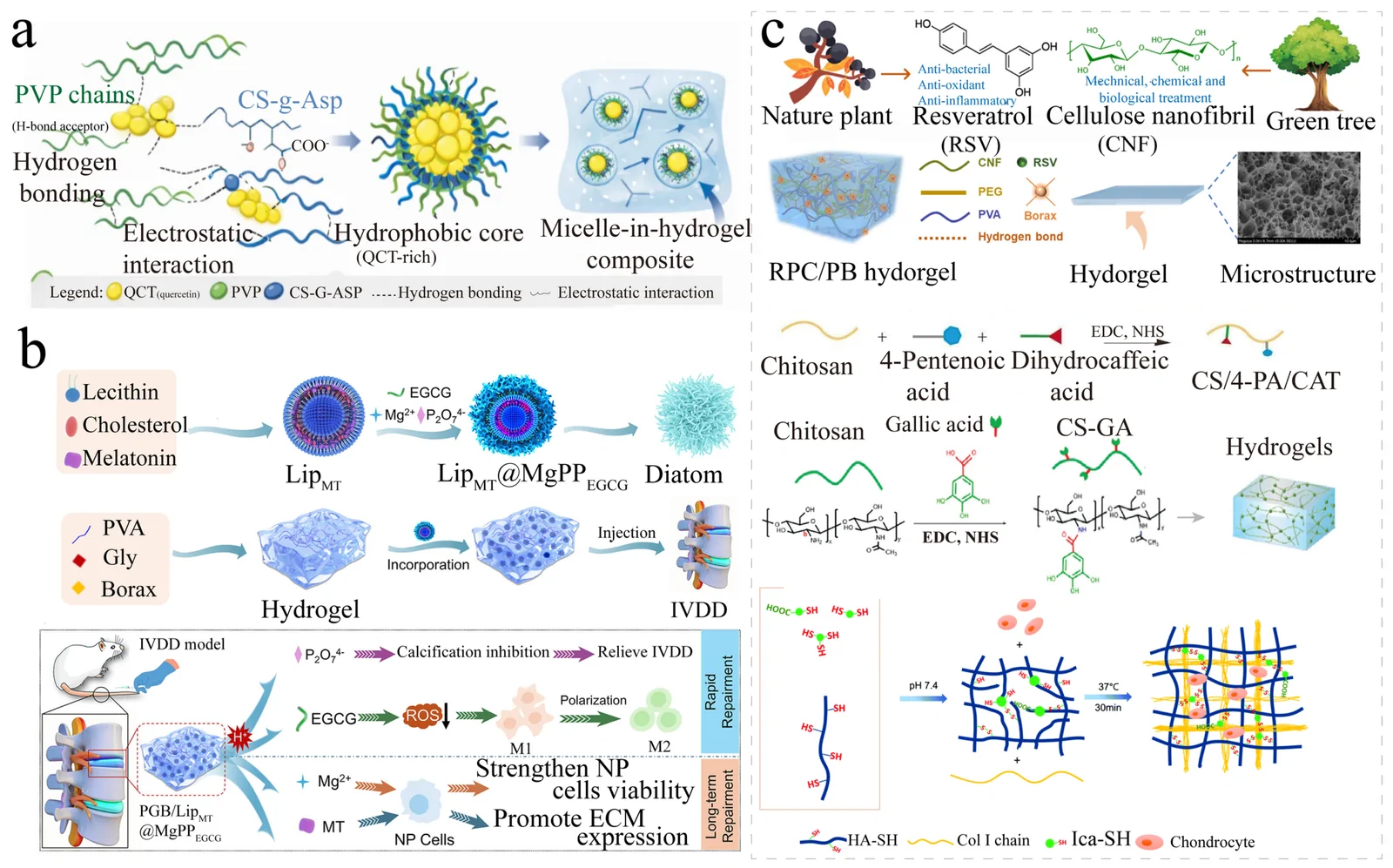
Representative composite-network strategies for incorporating TCM-derived bioactive components into hydrogel systems. (**a**) Quercetin-loaded polymeric micelles (QCT-M) incorporated into a thermosensitive hydrogel. Reproduced with permission from ref. [95]. Copyright 2026, Springer Nature. (**b**) Liposome-incorporated hydrogel designed to respond to the acid microenvironment for the treatment of intervertebral disc degeneration (IVDD). Reproduced with permission from ref. [92]. Copyright 2025, American Chemical Society. (**c**) Matrix-functionalized hydrogels based on poly(vinyl alcohol)-borax networks incorporating resveratrol-grafted cellulose nanofibrils, 4-glutenoic acid-modified chitosan injectable hydrogels, chitosan-gallic acid graft copolymers, catechol-functionalized chitosan-gallic acid graft copolymer for periodontal dressing hydrogel application, and thiolated icariin-functionalized HA/Col hydrogels. Reproduced with permission from ref. [105]. Copyright 2022, Springer Nature; ref. [107]. Copyright 2023, Elsevier; ref. [108]. Copyright 2024, Royal Society of Chemistry; ref. [110]. Royal Society of Chemistry.

One common route is a hierarchical carrier-in-carrier design. In this approach, nanoscale structures such as liposomes, micelles, mesoporous particles, nanoemulsions or exosome-like vesicles are first used to solubilize hydrophobic molecules, protect labile compounds or introduce responsiveness to intracellular or disease-associated cues [88,89,90,91]. These nanostructures are then embedded within a macroscopic hydrogel matrix, which provides local retention, barrier function, and conformability at the tissue interface (Figure 6a). Representative examples include nanoparticles loaded with TCM essential oils or poorly soluble flavonoids and subsequently integrated into polymer networks based on polyvinyl alcohol, polyethylene glycol diacrylate, gelatin methacryloyl, sodium alginate, chitosan, hyperbranched polyethylene glycol diacrylate, sodium carboxymethyl cellulose, Carbopol, hyaluronic acid or thermoresponsive polymers for localized therapy [11,92,93,94,95,96,97,98,99,100]. When both the embedded nanodomains and the surrounding hydrogel matrix respond to disease-relevant cues, such as ROS or enzymes, multistage release profiles can be achieved (Figure 7a,b) [92,95]. Ribeiro et al. dispersed curcumin-loaded mesoporous silica nanoparticles within a thermoresponsive hydrogel [99]. Hierarchical design should not be equated with intrinsic superiority. Increasing structural complexity also increases the demands placed on formulation control, manufacturing reproducibility, quality assessment and regulatory characterization. The value of these systems therefore depends less on the number of structural levels than on whether the added hierarchy produces a measurable improvement in pharmacokinetics, tissue exposure, local retention or therapeutic outcomes that justifies the additional translational burden. If a nanocomposite hydrogel provides no measurable advantage over a simpler formulation delivering the same total dose of the same active agent, the rationale for introducing the additional structural complexity becomes difficult to establish. Such comparisons remain important because hierarchical systems are often evaluated primarily on the basis of their structural sophistication, whereas the incremental benefit attributable to each additional level of organization is not always examined directly.

#### 3.2.2. Matrix-Functionalization Strategies

A complementary strategy is to modify the hydrogel matrix itself so that it can recognize, stabilize, or release TCM-derived molecules more selectively, without relying on a separate nanocarrier. These matrices can be engineered through dynamic covalent conjugation, including boronate ester bonds between polyphenols and polymer chains, incorporation of host motifs, interpenetrating networks, double-network architectures, nanocomposite reinforcement, and molecular imprinting [101,102,103,104,105,106,107,108,109,110,111,112]. Gallic acid-grafted chitosan provides a representative example. Here, gallic acid is covalently attached to chitosan through carbodiimide-mediated coupling, and the conjugate alone serves as the matrix-forming material (Figure 7c) [108]. Grafting introduces phenolic hydroxyl groups that contribute antioxidant and antibacterial activity, while the chitosan backbone provides mechanical integrity and hemostatic function. Liu et al. developed a thiolated icariin-functionalized collagen/hyaluronic acid hydrogel and assessed cartilage formation in vitro and in vivo (Figure 7c) [110]. Mohammadi et al. loaded a curcumin–hydroxypropyl-β-cyclodextrin inclusion complex into an alginate hydrogel [61]. Under the framework outlined in Box 1, a soluble inclusion complex incorporated into a conventional hydrogel is not considered a co-gelator. Unless the inclusion complex contributes to network connectivity or establishes a defined matrix–drug affinity, this system is more appropriately classified as matrix-assisted gelation rather than composite-network integration. It is therefore presented as a boundary case in Figure 5. The same distinction applies to medicinal polysaccharides. A TCM-derived polysaccharide that forms the continuous scaffold should be classified as a network-forming polymer rather than as a small-molecule gelator. Its use as a structural constituent of an engineered hybrid network falls within composite-network integration. Simply dissolving or dispersing it in an independently formed matrix does not establish participation in scaffold formation. Botanical origin alone is insufficient to establish a structural role.

Matrix functionalization offers a broader choice of molecular building blocks than purely self-assembled systems, with greater control over mechanical properties, processability, and disease-responsive behavior. These strategies are particularly valuable when direct self-assembly is difficult to achieve, when the active component requires protection or improved solubility, or when effective tissue-level performance depends on mechanical or interfacial properties that a purely supramolecular network cannot readily provide. By integrating the TCM-derived component into a pre-existing or engineered polymeric scaffold, these systems can therefore decouple some aspects of therapeutic loading from the requirements of network formation. However, the polymer matrix may also dilute or obscure the contribution of TCM-derived components. If the same polymer hydrogel without the TCM component performs equivalently in the relevant biological assays, then a therapeutic contribution from the TCM component has not been demonstrated under the conditions tested, regardless of its chemical presence in the network.

#### 3.2.3. Critical Comparison

Carrier-in-carrier designs and matrix functionalization address different formulation challenges. A separate carrier phase can accommodate a therapeutic compound without requiring covalent modification, but it also introduces additional interfaces and release processes. By contrast, matrix functionalization can retain an active group within or near the scaffold and regulate its interaction with the surrounding network. However, chemical modification may compromise molecular accessibility, biological activity, and the identity of the species released during degradation.

Mechanical improvements should also be considered in relation to the intended application. Reinforcement that improves handling and structural integrity may also affect injectability, erosion, or molecular transport through the network. Similarly, stronger drug–matrix interactions may prolong local retention but also reduce the availability of the drug to engage biological targets. The objective should therefore be to achieve an appropriate exposure profile rather than simply to minimize the release rate. Mechanistic interpretation becomes particularly challenging when both the carrier and the scaffold are bioactive. A complete formulation may outperform the free drug because of the contribution of the matrix, the embedded carrier phase, changes in local exposure, or a combination of these effects. Comparisons with blank matrices and drug-free carrier phases are therefore needed to separate these contributions. Likewise, pharmacological synergy should not be inferred solely from the superior performance of a multifunctional construct.

Manufacturing introduces further considerations. Hierarchical systems require control over particle composition, drug loading, size distribution, dispersion, and subsequent network formation. Functionalized matrices, in turn, require consistent control of chemical substitution, residual reagents, and the reproducibility of the resulting network. For both approaches, the effects of sterilization and storage on material structure, release behavior, and biological activity should be evaluated. Added complexity is justified only when it addresses a defined limitation that cannot be adequately resolved by a simpler formulation.

### 3.3. Matrix-Assisted Gelation of Multicomponent Formulations

Whereas self-assembly and composite-network integration emphasize materials design, matrix-assisted gelation of multicomponent formulations is more directly concerned with translational pharmaceutics. This route converts established TCM dosage forms, including tinctures, lotions, liniments, or topical liquids, into hydrogel systems intended to improve local pharmacokinetics, tissue conformity, usability, and patient adherence [113,114,115]. Conventional formulations may have short residence times, require frequent administration, cause vehicle-associated irritation, or provide limited control over local exposure. The active constituents do not necessarily participate in network formation. Instead, the entire multicomponent formulation is incorporated into—or gelled using—a gel-forming matrix with agents such as Carbopol 941 and xanthan gum [116] sodium carboxymethyl cellulose and glycerol [117], oxidized sodium alginate/carboxymethyl chitosan and chitosan quaternary ammonium salt [118], or carbomer [119]. Although this approach may offer less fundamental materials novelty than drug-as-material self-assembly, it can offer substantial value when evaluated by residence time, administration, local tolerability, dose retention, stability, and therapeutic consistency.

The key scientific question is therefore not whether such systems represent fundamental materials innovation, but whether they offer a meaningful translational advantage relative to the original dosage form. Evaluation should involve rigorous comparison with the original formulation, long-term stability studies and assessment of whether constituents undergo selective partitioning or degradation during storage and release. Any claimed improvement should be supported by pharmacokinetic or pharmacodynamic evidence where appropriate. Simply converting a formulation into a hydrogel is insufficient. The resulting design must address a specific and measurable limitation of the original formulation. An advantage does not necessarily require the introduction of a new molecular mechanism. For example, reducing the frequency of administration may improve adherence without changing the underlying mechanism of action. Such achievements should nevertheless be demonstrated using appropriate patient-level measures, and any claimed therapeutic improvement must be supported by relevant clinical or pharmacodynamic outcomes. Neither improved adherence nor better treatment outcomes can be inferred from the material’s physical properties alone.

### 3.4. Comparative Assessment of the Three Routes

Self-assembly, composite-network integration, and matrix-assisted gelation of multicomponent formulations represent the principal design strategies currently used for TCM-associated hydrogels. These approaches differ not only in how TCM-derived components are incorporated into hydrogel systems but also in the functions assigned to them. Depending on the formulation, a TCM-derived constituent may serve as a network-forming unit, a bioactive crosslinker, a component of an assembled nanodomain, a matrix-interacting ligand, or primarily a therapeutic payload within a conventional gel-forming matrix.

Self-assembly systems achieve the closest integration between therapeutic and structural functions, but often face challenges related to narrow gelation windows, limited mechanical strength, and uncertain physiological stability. Composite-network integration offers broader tunability and better control over mechanics, processability, and disease-responsive behavior, although at the cost of greater architectural complexity and more demanding manufacturing requirements. Matrix-assisted gelation of formulations is the most conservative route in terms of material novelty, yet it may present a practical route towards near-term clinical translation when the objective is to improve an established TCM preparation.

The comparison summarized in Table 2 is therefore organized around a set of practical questions. What functions does the TCM-derived constituent actually serve within the material? What is the minimum evidence required to support the role? What are the characteristic failure modes? What comparators are necessary to establish that the TCM component contributes to the observed performance? These questions are more informative than hierarchical rankings of design strategies. The optimal architecture depends on the particular limitation that the hydrogel is intended to address, as well as the pathological environment, required residence time, mechanical demands of the target tissue, desired exposure profile, and level of manufacturing complexity that can be practically supported.

## 4. Pathological Barriers and Disease-Matched Hydrogel Design

The applications of TCM-associated hydrogels are more informative when organized according to the pathological barriers that limit conventional therapeutics than by disease labels alone. Across disease settings, the dominant obstacles differ as some tissue interfaces impose rapid fluid clearance, some are dominated by inflammation, oxidative stress, or infection, and others demand simultaneous structural support and sustained biochemical regulation [122]. We therefore organize this section around four recurring pathological barriers that limit conventional therapeutics. These are rapid local clearance, oxidative and inflammatory microenvironments, structurally deficient regenerative niches, and the need for spatial confinement when systemic exposure constrains treatment. For each barrier, we consider the material functions required, the corresponding engineering strategies, and the evidence needed to establish that the proposed design addresses the relevant pathological constraint. Table 3 provides a summary of the barrier-matched design strategies discussed in this section.

A second organizing principle concerns the level of evidence. Physicochemical evidence of gel formation, in vitro biological activity, in vivo efficacy, pharmacokinetic data, safety assessment, and clinical evidence represent categories of validation. These categories are not interchangeable. Demonstrating gelation in a buffer together with in vitro antibacterial activity does not establish that the material will function in an infected wound. Similarly, improved wound closure in a mouse model cannot by itself establish clinical benefit in patients. In vivo efficacy and pharmacokinetic or biodistribution studies provide complementary information. Efficacy data indicate whether a biological benefit is observed, whereas exposure data help explain where, for how long, and at what concentration the active constituents are available. Throughout this section, representative studies are therefore considered in relation to the level of evidence they provide, with particular attention to instances in which the interpretation extends beyond what the experimental data directly support. The six-level evidence framework used throughout this review is summarized in Table 4.

### 4.1. Rapid Local Clearance

Many TCM-derived molecules are cleared rapidly from the administration site by tear turnover, mucus renewal, peristalsis, blood flow, or wound exudate. Such clearance limits tissue exposure and makes it difficult to sustain sufficient local concentrations. Hydrogels can act as a viscoelastic depot, prolonging retention and helping to control the spatial distribution of therapeutic molecules. The ocular surface illustrates this problem. Continuous tear turnover rapidly removes conventional eye drops after instillation. Mucoadhesive and in situ-forming hydrogels can extend precorneal residence, potentially increasing local exposure to anti-inflammatory or antioxidant agents such as curcumin, baicalin, quercetin, puerarin, ferulic acid, and epigallocatechin gallate [110,150,151,152,153,154,155,156]. A thermosensitive chitosan-based gel containing ferulic acid has been reported to sustain release at the corneal surface, while a puerarin-containing poloxamer/Carbopol in situ-gelling system prolonged precorneal retention in rabbits [123,124,125,127,128,131]. These studies provide physicochemical and in vivo evidence for sustained release or retention, although pharmacokinetic measurements distinguishing free drug from hydrogel-associated drug are often lacking.

**Figure 8:**
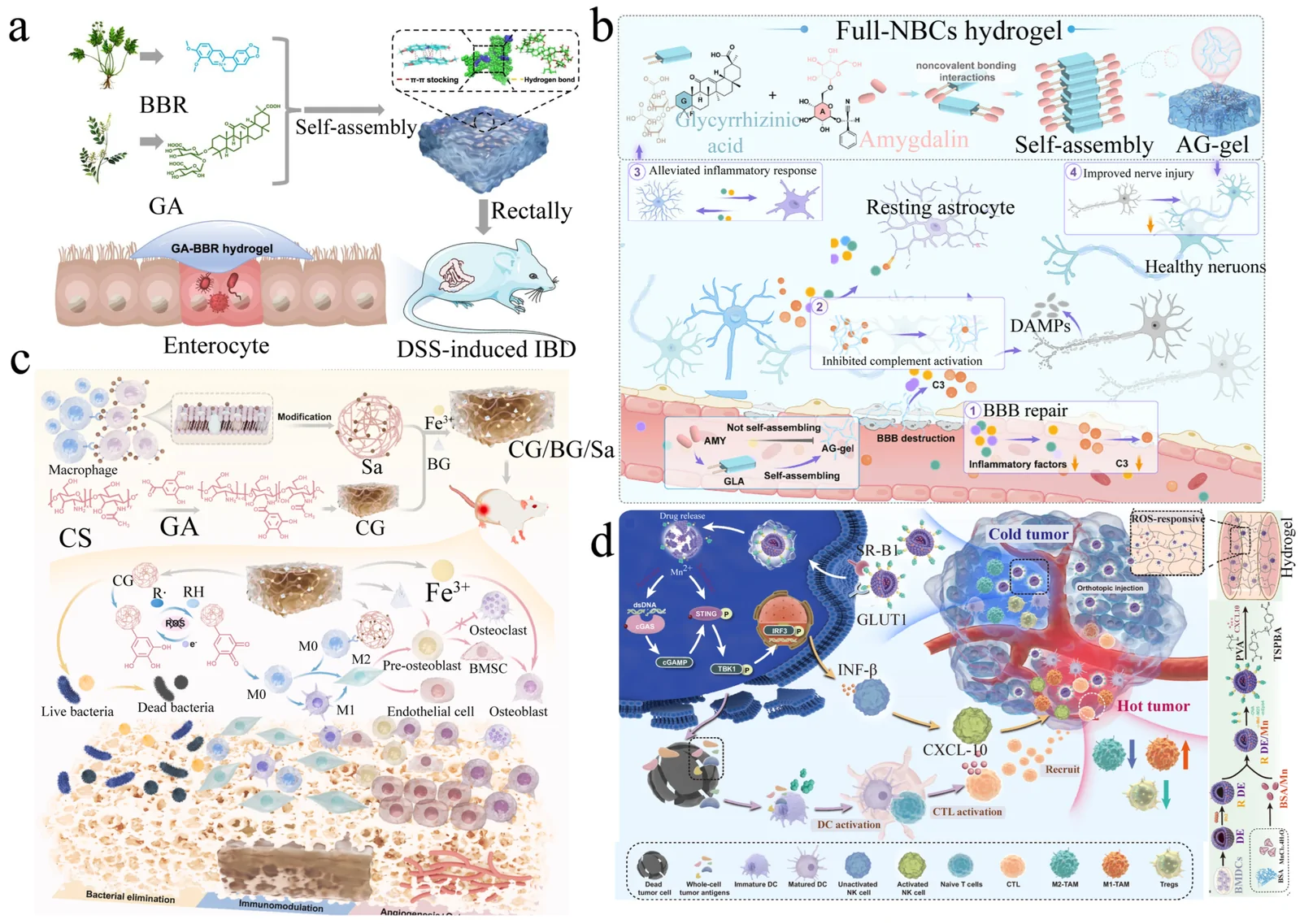
Disease-matched hydrogel design for overcoming pathological barriers. (**a**) Self-assembly hydrogel formed from berberine (BBR) and glycyrrhizic acid for the treatment of ulcerative colitis, illustrating the use of localized retention and mucosal exposure to address rapid clearance in the gastrointestinal tract. Reproduced with permission from ref. [52]. Copyright 2025, Elsevier. (**b**) In situ self-assembled hydrogel based on amygdalin (AMY) and glycyrrhizic acid (GLA) for administration to postoperative brain lesions and the treatment of traumatic brain injury, highlighting the need for local retention and tissue-compatible mechanics in neural interfaces. Reproduced with permission from ref. [12]. Copyright 2025, John Wiley and Sons. (**c**) Injectable composite hydrogel composed of gallic acid-modified chitosan (CG)/bioactive glass (BG)/sialic acid (Sa) for the repair of infectious bone defects, representing hydrogel design for structural support and regenerative microenvironment modulation. Reproduced with permission from ref. [140]. Copyright 2026, Elsevier. (**d**) Nanovaccine-loaded ROS-responsive hydrogel for reshaping the tumor microenvironment, illustrating locoregional hydrogel design for spatially confined tumor therapy. Reproduced with permission from ref. [143]. Copyright 2025, Springer Nature.

Intestinal diseases possess a more complex set of challenges. Therapeutic agents must withstand gastric conditions, resist enzymatic degradation, interact appropriately with the mucus layer, and remain sufficiently localized at sites of inflammation, which may change as the disease progresses. A ternary inulin hydrogel developed for inflammatory bowel disease was reported to prolong intestinal retention by exploiting the resistance of inulin to gastric digestion and its subsequent fermentation by colonic bacteria [157]. In a mouse model, the formulation reduced both intestinal inflammation and associated fibrosis, with these effects attributed to sustained local exposure and modulation of the gut microbiome. A self-assembled hydrogel formed from berberine and glycyrrhizic acid demonstrated improved intestinal retention compared with the free drug, based on fluorescence imaging of the Cy7-labeled hydrogel, and produced therapeutic efficacy in ulcerative colitis that exceeded that observed with mesalazine [52,157]. This system illustrates the application of the drug-as-material route to a disease setting in which local clearance limits exposure. However, persistence of the fluorescent signal does not by itself establish prolonged retention of either active compound or preservation of the assembled network, nor do therapeutic effects in a colitis model establish that both berberine and glycyrrhizic acid reach the inflamed tissue at sufficient concentrations to exert their effects. Component-resolved pharmacokinetic measurements in target tissue and plasma are needed to determine whether the therapeutic benefit is associated with local exposure, systemic exposure, or a combination of both. Mucosal adhesion and responsiveness to locally elevated ROS may help maintain therapeutic exposure at inflamed intestinal sites (Figure 8a) [52,157,158,159,160,161,162]. Across ocular and intestinal applications, the central design objective is to achieve sufficient local residence through an appropriate hydrogel architecture, with tissue- or mucosa-specific adhesion incorporated where appropriate. Matrix-assisted gelation and composite-network integration are widely used strategies for addressing this barrier. Drug-as-material systems may offer an alternative approach, provided that their local retention and physiological stability can be demonstrated. For these systems, evaluation should focus on direct measurements of residence time at the target site rather than relying solely on in vitro release profiles. A hydrogel that releases a compound slowly in phosphate-buffered saline (PBS) may still be rapidly cleared from the intestinal lumen if its interaction with mucosal is insufficient. The mismatch between in vitro release and in vivo retention remains a recurring limitation in this field.

### 4.2. Oxidative and Inflammatory Microenvironments

Many diseases, including chronic wounds, arthritis, and neural injury, are driven by persistent inflammation and oxidative stress [163,164]. Diabetic wounds, in particular, are characterized by substantially elevated ROS levels relative to healthy tissue, contributing to impaired angiogenesis and persistent macrophage-mediated inflammation. TCM-derived compounds often possess intrinsic antioxidant and anti-inflammatory activities, but the therapeutic relevance of these properties depends on maintaining adequate local exposure over an appropriate period.

The challenge in material design is therefore not simply to deliver an antioxidant compound, but to determine whether the hydrogel can intervene at multiple stages of the pathological process [165,166]. Metal–polyphenol networks are particularly interesting in this context because they can integrate ROS-scavenging activity, antimicrobial effects, and tissue adhesion within a single material architecture [159,167,168,169]. For example, a hydrogel formed through interactions among gallic acid, chitosan, and copper ions exhibited substantial ROS-scavenging activity and photothermal antibacterial effects against methicillin-resistant *Staphylococcus aureus* (MRSA), while also accelerating wound closure in an infected diabetic wound model [150,170]. In this system, gallic acid contributes to both network formation and antioxidant and antibacterial activity, providing an example in which the therapeutic and structural functions of a TCM-derived constituent are closely integrated.

The requirements are different in neural injury. Hydrogels must not only locally retain neuroprotective agents but also offer a mechanically matched environment that avoids secondary compression injury (Figure 8b) [12]. A self-assembled hydrogel based on rhein, berberine, and glycyrrhizic acid was developed for administration to postoperative brain lesions, with the objectives of local retention and tissue-compatible mechanics. The formulation improved functional recovery in a traumatic brain injury model relative to the free drug [86]. However, direct measurements comparing the hydrogel mechanics with those of brain tissue were not reported, nor was persistence of the assembled network at the lesion site established over time. These limitations make it difficult to determine whether the hydrogel architecture itself contributed substantially to the therapeutic outcome beyond improving local drug retention.

In arthritic joints, rapid clearance from the synovial cavity creates a related but distinct challenge. Here, the principal design objective is prolonged intra-articular exposure to anti-inflammatory agents. A sinomenine–glycyrrhizic acid self-assembled system enhanced the anti-inflammatory effects of sinomenine in a rheumatoid arthritis model, suggesting that co-assembly can improve therapeutic performance relative to the free compound [138]. The proposed mechanism involved improved solubility and sustained release provided by glycyrrhizic acid. However, the study did not compare the assembled hydrogel with an unassembled mixture containing equivalent amounts of the two components. Consequently, the specific contribution of co-assembly, as opposed to simple co-delivery, remains unresolved. Across oxidative and inflammatory applications, the most informative studies are those that combine multiple material functions and then establish which of those functions contributes to the biological outcome. A recurring weakness is treating sustained release as a mechanism in itself. Sustained release is a drug-delivery characteristic; its biological relevance must be demonstrated by linking the altered exposure profile to a specific therapeutic effect.

### 4.3. Structurally Deficient Regenerative Niches

In bone defects, cartilage lesions, and some soft-tissue injuries, hydrogels must support regeneration as well as deliver bioactive compounds. This requires temporary structural support, conformity to irregular defect spaces, and a microenvironment that permits cell infiltration, matrix deposition, and tissue remodeling (Figure 8c) [140]. In these settings, the therapeutic contribution of TCM-derived molecules depends substantially on sustained local exposure while the regenerative niche is being established. TCM-derived preparations and compounds, including red ginseng, resveratrol, and icariin, can modulate osteogenesis and osteoclast activity through pathways including Wnt/β-catenin and RANKL-RANK signalling [171,172]. Their value in hydrogel systems is less in simple delivery than in sustained presentation within a mechanically and biologically supportive matrix. For osteochondral defects, the spatial complexity of the bone–cartilage interface has encouraged the development of gradient or multilayered architectures [173,174]. A double-network bilayer hydrogel loaded with puerarin and curcumin was designed to address the different requirements of bone and cartilage, with the upper layer supporting chondrogenesis and the lower layer promoting osteogenesis [175]. These systems are designed to mimic the compositional and mechanical transition between cartilage and subchondral bone, and they generally rely on composite-network integration to combine structural support with spatially organized biochemical cues.

### 4.4. Spatial Confinement for Toxicity-Limited or Off-Target-Risk Lesions

Potent TCM-derived compounds may have a narrow therapeutic window because of poor solubility, rapid metabolism, systemic toxicity, or off-target exposure. Locoregional hydrogels can increase exposure at an accessible lesion while reducing, but not eliminating, systemic leakage. The clearest rationale is found in postoperative tumor beds, superficial tumors, and localized inflammatory lesions (Figure 8d) [143]. TCM-derived compounds with immunomodulatory, pro-oxidant, or chemosensitizing activities, including curcumin, tanshinone, and celastrol, have been incorporated into multimodal hydrogel systems together with chemotherapeutics, photothermal agents, or metal ions [1,143,144,146,176]. These systems are best viewed as tools for locoregional control or combination treatment rather than as substitutes for systemic therapy in disseminated disease. The benefit of spatial confinement should be judged by its effect on the therapeutic index. A hydrogel should maintain a clinically relevant drug concentration gradient at the lesion while limiting burst release, local toxicity, and systemic exposure. Assessment requires pharmacokinetic measurements of drug concentrations in target tissue and plasma over time, together with comparison against systemic administration of the same total dose. Many preclinical studies report improved efficacy without this direct comparison. In the absence of such data, the contribution of local delivery remains difficult to distinguish from the intrinsic potency of the formulation.

Local toxicity requires independent assessment. A cytotoxic or otherwise potent compound can injure tissue at high concentrations at the administration site, even when systemic exposure is low. Hydrogel degradation products may likewise provoke local inflammation. Assessment should therefore include local histopathology and inflammatory markers in addition to conventional systemic toxicity endpoints.

### 4.5. Cross-Barrier Comparison

Considering these four pathological barriers reveals several patterns that are less apparent when individual disease applications are examined separately. The first concerns the relationship between formulation complexity and the nature of the pathological barrier. Rapid-clearance problems, as encountered in ocular and intestinal delivery, are primarily pharmacokinetic. In many cases, a relatively simple mucoadhesive or in situ-forming matrix is sufficient to extend residence, making composite-network integration or matrix-assisted gelation appropriate without requiring drug-as-material self-assembly. Oxidative and inflammatory barriers are pharmacodynamic as well as pharmacokinetic. The material must remain at the pathological site while also modulating the local microenvironment. Metal–polyphenol networks and multifunctional co-assembled systems are therefore potentially useful when several therapeutic functions need to be integrated. Structurally deficient regenerative niches impose additional mechanical demands beyond those associated with retention and microenvironmental regulation. Here, the hydrogel must maintain space, withstand the relevant mechanical environment, and support tissue organization, making composite architectures with gradient or layered structures particularly relevant. In toxicity-limited lesions, the principal challenge is to establish a favorable spatial exposure profile while limiting systemic and local toxicity. Matrix-assisted gelation or composite-network systems with controlled release may therefore be sufficient, depending on the therapeutic objective.

The second pattern concerns the importance of experimental comparators. Across all four barriers, inadequate controls remain one of the most common limitations. A hydrogel that outperforms a free drug in a wound-healing model has not demonstrated that the specific hydrogel architecture is therapeutically important; the free compound may simply have been cleared too rapidly and a range of depot formulations could produce a similar result. Likewise, a hydrogel that outperforms a conventional drug-loaded hydrogel does not establish that the particular design feature under investigation, such as co-assembly, stimulus responsiveness, or hierarchical organization, is responsible for the improvement. The most informative comparator is therefore the same formulation with the feature under investigation removed or appropriately substituted.

The third pattern concerns the extent to which the available evidence supports claims of translational relevance. Many studies demonstrate therapeutic efficacy in rodent models and discuss clinical potential, but differences in immune responses, tissue architecture, and wound-healing kinetics can limit the extent to which these findings can be extrapolated to patients. Another limitation is that in vivo efficacy studies and pharmacokinetic studies are often reported as if they were interchangeable. They are not. A formulation may improve a disease endpoint in a rodent model while offering only transient or poorly distributed local exposure. Conversely, prolonged local residence does not necessarily translate into a therapeutic benefit. When a study claims that local delivery improved the therapeutic index, component-resolved pharmacokinetic data in target tissue and plasma are required, ideally alongside systemic administration at the same total dose. Without such data, the contribution of the hydrogel architecture to the observed efficacy remains uncertain. Large-animal models and human tissue explants can provide complementary evidence, but remain relatively uncommon in the literature. Translational maturity should therefore be assessed with these gaps in mind, as promising preclinical findings alone do not establish clinical benefit. Table 3 summarizes the design strategies used to address each barrier and the evidence required to evaluate their effectiveness.

## 5. Challenges and Future Directions

**Figure 9:**
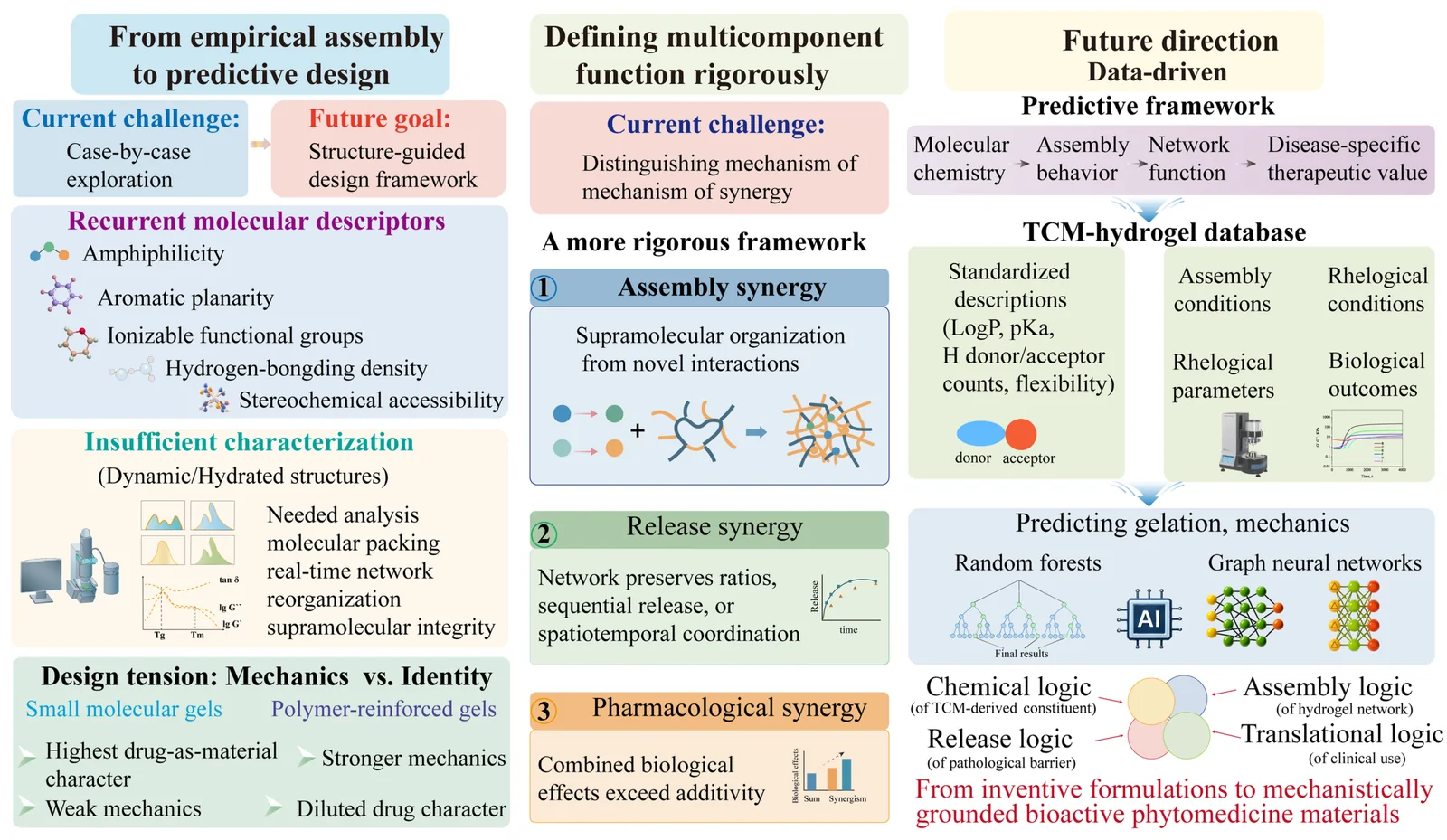
Key challenges and future directions of TCM-derived hydrogels.

Despite rapid progress, TCM-associated hydrogels are still making the transition from proof-of-concept formulations to predictive, translatable biomaterials. The central challenge is no longer whether such a hydrogel can be constructed, but whether its design logic is interpretable, reproducible, causally validated, and suited to a specific pathological context (Figure 9).

### 5.1. From Empirical Assembly to Predictive Design

One of the main challenges is the lack of a reliable structure-guided design framework. Gelators are still commonly identified on a case-by-case basis. Structural features such as amphiphilicity and stereochemical accessibility are often used to explain why a molecule forms a gel only after successful examples have been identified, rather than being incorporated into models that can predict gelation propensity, assembly pathways, or network behavior [177,178]. A more reliable structure–assembly framework should establish whether a TCM-derived molecule is likely to form a gel, the conditions required for gelation, and the properties of the resulting network. Developing such a framework will require comparable measurements across chemically diverse molecular families under carefully controlled experimental conditions. The recently proposed structure–assembly–property–function framework for TCM-derived monomers offers a useful conceptual starting point, although its current application remains largely descriptive. In principle, a quantitative model could, for instance, estimate the critical gelation concentration of a flavonoid based on calculated LogP, pKa, and hydrogen-bonding parameters. Machine learning could assist this work, but its practical application is currently constrained by the limited size and inconsistent quality of the available training datasets.

Material design must also take into account the balance between mechanical robustness and the drug-as-material principle. A supporting polymer can enhance mechanical stability, processability, and handling. As the amount of polymer reinforcement increases, however, the TCM-derived constituent may gradually function more as a therapeutic payload than as an integral structural element of the material. The appropriate balance will depend on factors such as the route of administration, the required residence time, and the mechanical environment of the target tissue. For example, a gel designed for the ocular surface may need only moderate mechanical strength, whereas a scaffold intended to support a bone defect would be expected to withstand substantially greater mechanical demands. The important consideration is therefore not whether polymer reinforcement is introduced, but whether its extent is appropriate for the intended application and whether the TCM-derived constituent continues to play a clearly defined structural or functional role in the final material.

Structural characterization also needs to extend beyond morphology and bulk mechanics. Electron microscopy and rheology provide information, but generally cannot resolve molecular packing, time-dependent network reorganization, or supramolecular integrity under physiological conditions. Structures that appear highly ordered in transmission electron microscopy (TEM) images may reorganize substantially upon exposure to serum proteins, salts, or other components of biological fluids. Time-resolved and in situ characterization are needed to follow assembly and network formation. Techniques such as small-angle X-ray scattering (SAXS), nuclear magnetic resonance (NMR) relaxometry, and fluorescence correlation spectroscopy (FCS) can provide complementary information on molecular organization and network dynamics under conditions closer to the physiological conditions.

### 5.2. Rigorous Definition of Multicomponent Function

Enhanced efficacy is frequently labelled “synergy”, even when it may result from prolonged local retention, modified release kinetics, increased solubility, or the additive effects of co-delivery. These contributions may improve treatment, but none alone establishes pharmacological synergy. A more rigorous analysis would distinguish at least three levels of multicomponent function. Structural cooperation occurs when several components produce an organization or network architecture that no individual component can achieve alone. Coordinated delivery occurs through the preservation of component ratios, the ordering of exposure over time, or improved release behavior relative to an unassembled mixture. Pharmacological synergy requires a combined biological effect that exceeds the additive response under controlled exposure conditions. Future studies should explicitly state which form of function is being claimed and provide corresponding evidence. For structural cooperation, this means molecular and microstructural characterization demonstrating the contributions of the individual components to the assembled architecture. For coordinated delivery, it means quantitative component-resolved release analysis. For pharmacological synergy, it means dose–response analysis and evaluation using an appropriate additivity model, with combination-index analysis where applicable. An additional test is to determine whether disrupting the assembled structure reduces or eliminates the disease-relevant benefit while controlling for changes in other formulation variables. Such a result supports an assembly-dependent contribution, but does not by itself establish pharmacological synergy. If disruption has no effect, an assembly-dependent benefit has not been demonstrated under the conditions tested. This framework is summarized in Table 5.

Greater attention is needed to distinguish structural from pharmacological contributions. In co-assembled systems, one component may strengthen the network while another provides therapeutic activity. Improved outcomes may stem solely from mechanical stabilization and prolonged local retention, rather than from biological interaction between the constituents. This is a valuable material contribution, but describing it as a pharmacological synergy obscures the mechanism and makes comparison across studies more difficult.

### 5.3. Reproducibility, Manufacturing and Translational Positioning

Reproducibility must be considered alongside manufacturability from the outset. In botanical systems, variation may arise at multiple stages, including the source material, harvesting conditions, extraction, purification, and storage. This can affect the composition of extracts and fractions. Thorough characterization is therefore essential, whether by high-performance liquid chromatography (HPLC), fingerprinting, or other appropriately validated profiling methods. Studies should quantify batch variability and state clearly whether the system is based on chemically defined molecules or on complex fractions or extracts [179]. Equivalent care is required at the formulation stage. Variables such as the order of mixing and the ionic environment may influence assembly and should be controlled and reported in detail. Researchers should establish whether particular constituents are preferentially incorporated into the network, protected from degradation, or lost during storage and release. This issue is particularly relevant to multicomponent formulations, in which relatively modest changes in component ratios may affect both pharmacological activity and material behavior. Manufacturing assessment should address whether the formulation can be prepared reproducibly, whether its critical quality attributes can be measured reliably, whether it is compatible with sterilization, and whether it remains stable over clinically relevant periods [180]. These requirements should inform formulation design at an early stage, rather than being treated as downstream technical considerations. The intended translational position of a formulation also needs clearer definition. A self-assembled gel composed entirely of active natural products presents a different set of scientific, manufacturing, and regulatory considerations from a traditional polymeric carrier containing a defined herbal extract. Both differ again from multifunctional nanocomposites that incorporate, for example, metal ions, synthetic nanocarriers, and immunomodulatory constituents. Some studies may be more valuable as mechanistic models for investigating structure–function relationships, whereas others may have a more direct role as advanced platforms for local delivery or modernized formulations of existing TCM preparations.

### 5.4. Data Infrastructure and Model Governance

Future progress is likely to depend less on increasing structural complexity than on improving interpretability, validation, and the matching of formulations to specific disease settings. Predictive design will require data that connect molecular chemistry to assembly, assembly to network function, and network function to therapeutic performance with a clearly defined pathological context. The chemical diversity of TCM-derived constituents, together with the interactions within multicomponent systems, makes this field well suited to data-driven investigation. A dedicated TCM-Hydrogel Database could provide a useful foundation by recording standardized molecular descriptors with experimental outcomes. Relevant descriptors would include LogP, pKa, hydrogen-bond donor and acceptor counts, aromaticity, ionization state, and conformational flexibility. These should be associated with assembly conditions, rheological parameters, release profiles, and biological outcomes [181,182]. Machine learning models, including random forests, gradient-boosting methods, or graph neural networks, could then be trained to predict gelation propensity, critical gelation concentration, mechanical behavior, and promising component combinations [183,184]. Such models may also help distinguish different forms of multicomponent synergy by integrating structural, kinetic, and biological datasets. In this way, empirical trial-and-error could progressively give way to a more rational and accelerated design framework. The most convincing systems will not be those with the largest number of components or stimuli-responsive triggers, but those in which four forms of logic are brought into alignment, the chemical logic of the TCM-derived constituent, the assembly logic of the hydrogel network, the release logic required by the pathological barrier, and the translational logic of the intended clinical use. If this coherence can be achieved, with increasing support from machine-learning tools, TCM-associated hydrogels may develop from a collection of inventive formulations into a distinctive and mechanistically grounded class of bioactive biomaterials.

### 5.5. Safety and Translational Risk

Safety has received insufficient scrutiny in much of the literature on TCM-associated hydrogels. Natural origin does not establish safety, and hydrogel formulation may introduce risks beyond those associated with the free drug. Complex herbal preparations are particularly difficult to assess because their composition varies across batches, and minor constituents may have poorly characterized biological activity. Heavy metals, pesticide residues, mycotoxins, and microbial contamination may persist in the material. Chemical fingerprinting should therefore be combined with contaminant and microbial testing. Metal-containing hydrogels require assessment of both the network and metal exposure. Metal–polyphenol and other coordination networks are promising, but excessive ion release may cause cytotoxicity. Assessment should therefore encompass dose-dependent toxicity, local and systemic exposure, release kinetics, organ accumulation, in vivo metabolism, and long-term biosafety. Coordination-dependent release and metal-specific toxicity also carry implications for regulatory positioning. Reports of good tolerability in animals should not be generalized across zinc, iron, cerium, silver, and copper, as these metals have distinct toxicological profiles. Degradation and long-term biocompatibility also require evaluation in multicomponent hydrogels. Products from synthetic scaffolds may trigger inflammatory or immune responses, whereas TCM-derived constituents may exert unanticipated effects on surrounding tissue. Studies that extend beyond short-term rodent experiments are therefore needed to examine chronic inflammation, fibrous capsule formation, and systemic accumulation of degradation products. Local safety must likewise be assessed independently of systemic toxicity. Even when systemic exposure is limited, high concentrations at the administration site may cause necrosis, inflammation, or pain. Such effects may be overlooked where safety assessment relies mainly on systemic toxicity measures. Table 6 provides a summary framework linking material architecture, mechanism, therapeutic application, evidence levels reported in representative studies, and the principal translational limitation. The evidence levels are ranges observed in the cited studies, not as intrinsic rankings of the material architectures. Representative references are given in the table so that each assignment can be traced back to the primary literature. As discussed in Section 4, in vivo efficacy and pharmacokinetic or biodistribution evidence are complementary rather than hierarchical.

## 6. Conclusions

TCM-associated hydrogels are most compelling when their therapeutic chemistry and material architecture are mechanistically coupled. The three engineering routes of drug-as-material self-assembly, composite-network integration, and matrix-assisted gelation of multicomponent formulations help to clarify both the scientific contribution and the translational burden of individual systems. Matching these routes to specific pathological barriers further shifts evaluation from formulation novelty to disease-relevant function. Key priorities include predictive structure–assembly relationships, causal validation of multicomponent effects, component-resolved quality control, physiological stability, scalable manufacturing, and clear regulatory positioning. Successful designs must align the chemistry of the TCM-derived constituent with gel-network assembly, the exposure profile required to address the relevant pathological barrier, and the practical requirements of clinical use. Machine learning may support this transition by informing design choices and helping to prioritize experiments, but it cannot substitute for rigorous mechanistic and translational validation. Together, these advances could establish TCM-associated hydrogels as a distinctive class of bioactive biomaterials with a clear mechanistic rationale.

## Figures and Tables

**Table 1:** Key molecular interactions governing the self-assembly of TCM-associated hydrogelators.

Molecular Interaction	Structural Requirements in TCM-Derived Molecules	Representative Molecular Classes	Suggested Validation Methods
π-π stacking	Planar or partially planar aromatic domains with suitable electronic distribution and packing geometry. Excessive planarity may favor crystallization rather than gelation.	Flavonoids and anthraquinones [32,33,34,35], phenolic acids [35,36], alkaloids and other aromatic natural products [37].	UV–visible spectroscopy, fluorescence spectroscopy, circular dichroism, X-ray scattering, microscopy and comparison with structurally related analogues lacking aromatic planarity
Hydrogen bonding	Hydrogen-bond donors and acceptors, including hydroxyl, carboxyl, carbonyl, amide, glycosidic or phenolic groups, arranged with suitable spatial accessibility.	Saponins [38], flavonoids [39], phenolic acids, polyphenols, polysaccharides and glycosides [40]	Fourier-transform infrared spectroscopy, variable-temperature spectroscopy, rheology, solvent competition experiments and gelation tests in biologically relevant media.
Hydrophobic association	Hydrophobic domains balanced by hydrophilic groups, producing amphiphilicity sufficient for aggregation without macroscopic phase separation	Saponins [38,41], flavonoids [42], amphiphilic glycosides and hydrophobic natural products [43,44].	Critical aggregation concentration, fluorescence probe assays, dynamic light scattering, cryo-electron microscopy, rheology and serum-stability tests.
Electrostatic interactions	Ionizable groups such as protonated amines, carboxylates, phenolates, sulfates or phosphates with complementary charge distribution.	Triterpenoid acids [45,46], alkaloids, saponins and hydrophobic or monoterpenoid phenols [47], triterpenoid saponins [48,49], flavonoids and isoflavones [50,51], alkaloids [14].	Zeta-potential analysis, pH-dependent gelation studies, salt-resistance tests, isothermal titration calorimetry, rheology under physiological ionic strength and component-ratio tracking.
Metal–ligand coordination	Ligand groups capable of coordinating metal ions, including catechol, phenolic hydroxyl, carboxylate or nitrogen-containing groups.	Flavonoids and flavonoid glycosides [52,53,54,55], glycyrrhizic acid [56,57], triterpenoid acids [58,59], phenolic acids [60].	UV–visible spectroscopy, X-ray photoelectron spectroscopy, rheology, metal-ion titration, pH-dependent stability tests, cytotoxicity assays and metal-release measurements.
Host–guest recognition	Complementary host and guest motifs with suitable binding affinity, reversibility and aqueous compatibility. Host molecules are often introduced through cyclodextrins, calixarenes, cucurbiturils.	(2-hydroxypropyl)-β-cyclodextrin [61,62], TCM-derived active component-β-cyclodextrin inclusion complexes [63,64,65,66].	Nuclear magnetic resonance spectroscopy, isothermal titration calorimetry, competitive binding assays, rheology, release studies and comparison with non-complexed formulations.
Van der Waals and cooperative weak interactions	Close molecular packing enabled by complementary shape, conformational flexibility and appropriate molecular volume. These interactions rarely act alone but cooperate with stronger directional interactions.	Broadly present across small molecules, glycosides, terpenoids, alkaloids, flavonoids and polysaccharides [38,45,51,67].	Molecular simulation, temperature-dependent rheology, structure–activity comparison and combined analysis with spectroscopy, scattering and microscopy.

Table note. Detailed failure modes for each interaction are provided in Supplementary Table S2. The table is intended to guide experimental design rather than to serve as an exhaustive catalogue. Many TCM-associated hydrogelators assemble through combinations of these interactions, and the dominant driving force may shift with concentration, pH, ionic strength, and temperature.

**Table 2:** Comparison of the three engineering routes for TCM-associated hydrogels.

Dimension	Drug-as-Material Self-Assembly	Composite-Network Integration	Matrix-Assisted Gelation
Defining design logic	Individual TCM molecule forms a supramolecular gel through its intrinsic intermolecular interactions. The therapeutic molecule is also the primary structural unit.	TCM-derived components are incorporated into an engineered polymer network, providing bioactivity and contributing to crosslinking, adhesion, reinforcement, or responsiveness.	Complete TCM formulation is converted into a hydrogel dosage form to improve local retention, tissue conformity, dosing convenience, and patient compliance.
Role of TCM component	Principal gelator or co-gelator and therapeutic component, the active contribute to network formation, local presentation, degradation behavior, and biological activity.	Network-forming or network-modifying component, dynamic crosslinker, interfacial modifier, antioxidant, antimicrobial agent, or therapeutic payload within an engineered composite architecture, polymer matrix remains the main structural scaffold.	Therapeutic ingredient rather than network-forming unit, hydrogel acts as a pharmaceutical vehicle.
Typical interactions or gelation mechanisms	Hydrogen bonding, hydrophobic association, π–π stacking, electrostatic interactions, metal–ligand coordination, pH-dependent amphiphilic assembly.	Dynamic covalent bonds, boronate ester linkages, Schiff-base reactions, metal–phenolic coordination, hydrogen bonding, host–guest interactions, electrostatic complexation, interpenetrating or double networks.	Physical gelation, ionic crosslinking, or viscosity enhancement using carbomer, alginate, chitosan, gelatin, cellulose derivatives, or poloxamers.
Representative systems	Glycyrrhizic acid [56], Zingibroside R [69], puerarin [50], quercetin [73], rhein [75], Berberine–glycyrrhizic acid systems [120], glycyrrhizic acid–rhein–berberine combinations [86].	Gallic acid-grafted chitosan [101], catechol-modified chitosan [107], thiolated icariin-HA [110], resveratrol–cellulose nanofiber-PVA-borax [105]	TCM extracts, decoction-derived preparations, tinctures, lotions converted into gel formulations [116,121].
Main advantages	Close integration of therapeutic and structural functions, high drug-loading capacity, and the possibility of forming networks without a separate carrier matrix.	Broader molecular scope, improved mechanical strength, processability, injectability, and tissue adaptability, allows independent tuning of modulus, degradation, release, and bioactivity.	Directly connected to clinically familiar dosage forms, improves retention, spreadability, local exposure, and dosing convenience, attractive for near-term translation.
Key limitations	Narrow gelation windows, limited mechanical strength, uncertain physiological stability, difficult to predict assembly from molecular structure.	Polymer matrix may dilute or obscure TCM component contribution, increased formulation complexity complicates mechanism, sterilization, scale-up, and regulatory characterization.	Lower material novelty, hydrogelization may alter release, degradation, or efficacy of individual constituents, extract complexity complicates quality control.
Minimum validation	Demonstrate that the TCM molecule alone forms the network, compare assembled state with monomer solution at equivalent dose, assess stability in biologically relevant fluids.	Define whether TCM compounds act as payload, crosslinker, reinforcement domain, or interfacial modifier, compare with same polymer hydrogel without TCM component, quantify mechanics, adhesion, degradation, and release.	Use original TCM formulation as key comparator, chemically fingerprint formulation before and after hydrogelization, track representative marker components, demonstrate measurable improvement in retention, dosing, or therapeutic consistency.
Characteristic failure modes	Molecule crystallizes or precipitates instead of gelling, gel forms in buffer but disassembles in biological fluids, therapeutic effect indistinguishable from monomer solution.	Added complexity does not improve outcomes relative to simpler hydrogel, TCM component contribution cannot be isolated from polymer matrix effects.	Improved efficacy reflects prolonged contact rather than new material functions, component ratios change during storage or release.

Table note. Systems that combine host–guest recognition with polymer scaffolds are classified according to their dominant network architecture, as described in Box 1 and Section 3.1.2. Host–guest recognition alone is not enough to classify a system as multicomponent co-assembly. Detailed validation requirements, including recommended additional experiments and essential controls, are provided in Supplementary Table S3. The three routes are not mutually exclusive; a single system may combine elements of co-assembly and polymer reinforcement. The classification is operational and is intended to clarify what a given system can demonstrate about the relationship between material architecture and therapeutic function.

**Table 3:** Barrier-matched design of TCM-associated bioactive hydrogels for pathological tissue interfaces.

Pathological Barrier	Representative Disease Settings	Preferred Engineering Routes	Representative TCM-Derived Components or Formulations	Minimum Validation Required
Rapid local clearance	Ocular disease, oral ulcers, intestinal inflammation.	Matrix-assisted gelation. Composite-network integration	Curcumin [123], baicalin [124], puerarin [125], resveratrol [126], ferulic acid [127], epigallocatechin gallate [128], rutin [129], quercetin [130,131], hesperidin [132], rosmarinic acid [133], berberine and glycyrrhizic acid [52]	Direct measurement of in vivo retention (not only in vitro release); comparison with free drug at equivalent dose.
Oxidative/inflammatory microenvironments	Chronic wounds, arthritis, neural injury	Metal–polyphenol networks, multicomponent co-assembly, composite-network integration.	Glycyrrhizic acid [134], berberine and rhein [86], icariin [135] amygdalin [12], Rhein [33], sodium aescinate [49]	Comparison with non-antioxidant hydrogels and free drug, demonstration that ROS modulation contributes to therapeutic effect.
Structurally deficient regenerative niches	Bone defects, cartilage defects, osteochondral interfaces	Composite-network integration, gradient or layered architectures.	Gypsogenin [136], Lotus root extract [137], Sinomenine-glycyrrhizic acid [138], curcuma [139], TCM-derived polysaccharides and pro-regenerative herbal extracts, combined with matrices such as gelatin, chitosan, alginate, hyaluronic acid, collagen-mimetic networks or mineralized composite hydrogels [140,141,142]	In vivo functional assessment, mechanical testing of regenerated tissue, long-term stability.
Spatial confinement for toxicity-limited or off-target-risk lesions	Post-surgical tumor beds, superficial tumors, localized inflammatory lesions.	Composite-network integration, matrix-assisted gelation.	Melittin [143], celastrol [144], cordyceptic acid [145], glycyrrhizic acid and lycorine [146], elemene [88], gambogic acid [147], paclitaxel-related natural products [148], honokiol [149]	Pharmacokinetic comparison with systemic administration, local histology at administration routes site, dose–response for efficacy and toxicity.

Table note. Evidence levels for representative studies are provided in Supplementary Table S1. Preferred engineering routes are indicated based on the dominant barrier in each disease setting, individual systems may combine routes. Minimum validation requirements are intended as a baseline, rather than an exhaustive list. Translational cautions specific to each barrier are discussed in the main text.

**Table 4:** Evidence framework for TCM-associated bioactive hydrogels.

Level	Evidence Type	Required Experiments	Translational Implication
Level 1	Physicochemical proof of gel/network formation	Rheology (G′, G″), SAXS, TEM/SEM, FTIR, X-ray scattering	Establishes material feasibility but not therapeutic relevance
Level 2	In vitro biological activity	Cell assays (cytotoxicity, proliferation, differentiation), antimicrobial, antioxidant, anti-inflammatory assays	Demonstrates bioactivity but not in vivo efficacy, does not account for clearance, metabolism, or tissue penetration
Level 3	In vivo efficacy	Animal models of disease, histological analysis, functional recovery assessment	Supports therapeutic potential but not clinical translation, species differences limit predictive value
Level 4	Pharmacokinetic/biodistribution	Fluorescence or isotope labeling, ICP-MS, component-resolved pharmacokinetics	Establishes local versus systemic exposure profiles, necessary for comparing local delivery with systemic administration
Level 5	Safety/toxicology	Cytotoxicity, hemocompatibility, long-term implantation, histology, inflammatory cytokine measurements at administration site	Essential prerequisite for clinical consideration; local toxicity can occur without systemic toxicity
Level 6	Clinical evidence	Randomized controlled trials, real-world studies	Required for clinical adoption, currently absent for most TCM-derived hydrogel systems.

Table note. This table is intended to clarify the scope of claims supported by each evidence level, rather than to assign overall quality rankings to individual studies. A study that reports only Level 1 or Level 2 evidence cannot substantiate claims about therapeutic efficacy. A study that reports Level 3 evidence in a rodent model cannot by itself establish clinical benefit or readiness for clinical application. Level 3 and Level 4 evidence is complementary rather than hierarchical. In vivo efficacy does not establish local or systemic exposure, and pharmacokinetic or biodistribution data do not establish therapeutic benefit. Claims of local delivery advantage require both exposure data and disease-relevant outcomes, ideally with comparison to systemic administration at the same total dose. The framework is applied in Supplementary Table S1, which includes an “Evidence level” column for each catalogued system, and in the discussion of individual applications throughout Section 4.

**Table 5:** Distinguishing structural cooperation, coordinated delivery, and pharmacological synergy.

Functional Level	Definition	Required Experimental Evidence	Common Misattribution
Structural cooperation	Multiple components form an organization or network that cannot be achieved by any single component alone	NMR, ITC, CD, SAXS, rheology, microscopy, comparison with individual components and physical mixtures	Attributing performance improvement of simple mixtures to co-assembly
Coordinated delivery	The network preserves component ratios, coordinates sequential exposure, or outperforms an unassembled mixture in release behavior	Component-resolved release kinetics, ratio preservation analysis, spatial distribution mapping	Equating altered release behavior with synergy
Pharmacological synergy	Combined biological effects exceed additivity under controlled exposure	Dose–response curves, combination index, isobologram analysis, unassembled mixture controls, mechanistic pathway validation	Equating increased local concentration with true pharmacological synergy.

**Table 6:** Summary framework for TCM-associated bioactive hydrogels.

Material Architecture	Mechanism	Therapeutics Application	Evidence Levels Reported in Representative Studies	Representative Studies	Key Translational Limitation
Single-mole self-assembly	Network formation through intrinsic intermolecular interactions	Neural inflammation, wound healing, antibacterial therapy.	Level 1–3	Glycyrrhiz acid [185], rhein [33], quercetin [73]	Narrow gelation window, uncertain physiological stability
Multicomponent co-assembly	Cooperative assembly for structural reinforcement and/or controlled release.	Ulcerative colitis, wound healing, arthritis	Level 2–4	Berberine-glycyrrhizic acid [52], berberine-glycyrrhizic acid-rhein [86], sinomenine-glycyrrhiz acid amygdalin-glycyrrhizic [138]	Difficulty to distinguish co-assembly from simple co-delivery
Metal–polyphenol network	Coordination crosslinking with ROS-scavenging and antibacterial activity.	Infected wounds, diabetic ulcers	Level 2–4	Gallic acid-chitosan-Cu^2+^ [150], baicalin-Zn^2+^ [77], rhein nanozyme [78]	Metal release kinetics, long-term biosafety
Hierarchical carrier-in-carrier	Nanocarriers embedded within a macroscopic hydrogel depot	Cancer, intervertebral disc degeneration	Level 2–3	Quercetin-ZIF-8/carboxymethyl chitosan [87], curcumin-mesoporous silica nanoparticle thermogel [99]	Added complexity without clear benefit over simpler systems
Matrix-functionalized polymer	Covalent or coordinated-based incorporation of TCM components into a polymer matrix.	Bone regeneration, wound healing	Level 2–4	Gallic acid-grafted chitosan [101], thiolated icariin-HA/Col [110], resveratrol-cellulose [105]	TCM component contribution may be obscured by polymer matrix
Matrix-assisted gelation of formulation	Conversion of established TCM dosage form into a hydrogel.	Ocular, topical, mucosal applications	Level 1–2	Ferulic acid-chitosan [186,187], puerarin-poloxamer/Carbopol [125], curcumin-HPβCD [188]	Lower material novelty, quality control of complex extracts.

Table note. Evidence levels follow the six-level framework in Table 4. The levels reported here reflect the range of evidence described in the cited representative studies; they should not be interpreted as intrinsic rankings of the material architectures. A given study may contribute evidence at more than one level. Level 3 (in vivo efficacy) and Level 4 (pharmacokinetic and biodistribution) address complementary aspects of evaluation. Demonstration of in vivo efficacy alone does not establish local or systemic drug exposure, while pharmacokinetic or biodistribution data alone do not demonstrate therapeutic benefit. Claims of local delivery advantage should therefore be supported by both exposure data and disease-relevant outcomes, preferably against systemic administration at the same total dose. The table is intended as a summary index rather than a substitute for the detailed discussion in Section 3, Section 4 and Section 5. Not every system falls neatly into a single category. Hybrid architectures that combine co-assembly with polymer reinforcement, for example, may span two rows. The principal translational limitation listed for each architecture is a commonly reported obstacle in the current literature, rather than an inherent property of the approach.

## Data Availability

The original contributions presented in the study are included in the article. Further inquiries can be directed to the corresponding authors.

## Supplementary Materials

The following supporting information can be downloaded at: https://www.mdpi.com/article/10.3390/gels12100937/s1, Table S1: Catalog of TCM-derived bioactive hydrogels organized by engineering route; Table S2: Molecular interactions governing self-assembly of TCM-derived hydrogelators: structural requirements, failure modes, and validation methods; Table S3: Detailed validation requirements for the three engineering routes; Table S4: Checklist for physiologically relevant characterization of TCM-associated hydrogels.
