# Supplementary material for: Traditional Chinese Medicine-Associated Bioactive Hydrogels: Material Roles, Network Design, and Therapeutic Applications
Source: Gels. 2026 Oct 20;12(10):937. doi: 10.3390/gels12100937 (PMC13704119; doi:10.3390/gels12100937)
Supplement: Supplementary file 1 [file gels-12-00937-s001.zip › gels-4596598-supplementary.pdf]

Abbreviation
traditional Chinese medicine: TCM
reactive oxygen species: ROS
high-performance liquid chromatography: HPLC
nuclear magnetic resonance: NMR
transmission electron microscopy: TEM
scanning electron microscopy: SEM
fluorescence correlation spectroscopy: FCS
isothermal titration calorimetry: ITC
fourier transform infrared spectroscopy: FTIR
methicillin-resistant Staphylococcus aureus: MRSA
circular dichroism: CD
small-angle X-ray scattering: SAXS

Supplementary Methods S1. Date-specific search strategies

Web of science Core Collection
Search string:
#1 Topic 1 = ("traditional Chinese medicine" or "TCM" or "herbal" or "natural product" or "botanical*")
#2 Topic 2 = ("hydrogel*" or "gel*" or "supramolecular hydrogel*" or "in situ gel*" or "injectable hydrogel*" or self-assembl* hydrogel*")
#3 Topic 3 = ("wound*" or "inflammation" or "regeneration" or "cancer" or "tumor" or "arthritis" or "ulcerative colitis" or "bone defect*" or "cartilage defect*" or "neural injury" or "traumatic brain injury" or "ocular" or "mucosal")
#4 Topic 4 = ("glycyrrhizic acid" or rhein or berberine or curcumin or quercetin or baicalin or puerarin or icariin) and #2 Topic 2
Refine Publication Years = (2010-2026) Language: English Comments, editorials, letters, books, and conference abstracts indexed were excluded
Scopus
Search string:
#1 Article title, Abstract, Keywords = ("traditional Chinese medicine" or "TCM" or "herbal" or "natural product" or "botanical")
#2 Article title, Abstract, Keywords = ("hydrogel*" or "gel*" or "supramolecular hydrogel*" or "in situ gel*" or "injectable hydrogel*" or self-assembl* hydrogel*")
#3 Article title, Abstract, Keywords = ("wound*" or "inflammation" or "regeneration" or "cancer" or "tumor" or "arthritis" or "ulcerative colitis" or "bone defect*" or "cartilage defect*" or "neural injury" or "traumatic brain injury" or "ocular" or "mucosal")
#4 Article title, Abstract, Keywords = ("glycyrrhizic acid" or rhein or berberine or curcumin or quercetin or baicalin or puerarin or icariin) and #2
Refine Publication Years = (2010-2026) Language: English, Comments, editorials, letters, books, and conference abstracts indexed were excluded.
PubMed
Search string:

#1 ("traditional Chinese medicine" or "TCM" or "herbal" or "natural product" or "botanical")
#2 ("hydrogel*" or "gel*" or "supramolecular hydrogel*" or "in situ gel*" or "injectable hydrogel*" or self-assembl* hydrogel*")
#3 ("wound*" or "inflammation" or "regeneration" or "cancer" or "tumor" or "arthritis" or "ulcerative colitis" or "bone defect*" or "cartilage defect*" or "neural injury" or "traumatic brain injury" or "ocular" or "mucosal")
#4 ("glycyrrhizic acid" or rhein or berberine or curcumin or quercetin or baicalin or puerarin or icariin) and #2
Refine Publication Years = (2010-2026) Language: English Comments, editorials, letters, and conference abstracts indexed were excluded.
Record count table for filling in actual numbers

Stage	Web of Science	PubMed	Scopus
Records identified	7993	4788	15849
Duplicates removed		6874	
Title/Abstract screened		4427	
Eligible studies		1543	
Prioritized for detailed discussion		190	
Cited as supporting or comparator evidence		18	

Supplementary Table S1. Catalog of TCM-derived bioactive hydrogels organized by engineering route.

Engineering route	TCM source	Active component	Hydrogel system	Gelation/network mechanism	Roles of TCM component	Therapeutic application	Evidence level	Key limitation
Drgu-as-material self-assembly	Glycyrrhiza glabra	Glycyrrhizic acid	Single-component supramolecular hydrogel	pH-dependent hydrogen bonding and hydrophobic association; helical nanofibers	Primary gelator and therapeutic component	Neuroinflammation, wound healing, antibacterial therapy	Level 1-3	Narrow assembly window, limited mechanical strength, and uncertain stability in physiological fluids
	Rhubarb	Rhein		π–π stacking, pH-dependent carboxyl protonation		Neural inflammation; osteoarthritis	Level 1-3	Concentration- and solvent-dependent assembly, limited mechanical strength
	Pueraria lobata	Puerarin		Hydrogen bonding, aromatic stacking		Hydrogen bonding, aromatic stacking	Level 1-2	Slow gelation, mechanical fragility
	Various plants	Quercetin		Hydrogen bonding, π–π		Diabetic wound healing, tissue	Level 1-3	Poor water solubility,

			stacking, hydrophobic association		engineering		narrow assembly window
Scutellaria baicalensis	Baicali	Metal-coordinated or dynamic covalent hydrogel	Metal-ligand coordination, hydrogen bonding	Primary gelator or bioactive crosslinker	Antibacterial, antioxidant	Level 1-2	Sensitivity to metal ion stoichiometry
Coptis chinensis+Glycyrrhiza glabra+rhubarb	Berberine+glycyrrhizic acid+rhein	Ternary co-assembled hydrogel	Cooperative hydrogen bonding, π–π stacking, electrostatic and metal coordination	Co-assembling components, structural and therapeutic roles	Traumatic brain injury	Level 2-3	Mechanistic contribution of each component not fully isolated
Sinomenium acutum+Glycyrrhiza glabra	Sinomenine+glycyrrhizic acid	Co-assembled hydrogel	Hydrogen bonding, hydrophobic association, electrostatic interaction	o-assembling components, GA improves solubility	Rheumatoid arthritis	Level 2-3	No unassembled mixture control; synergy not established
Prunus dulcis + Glycyrrhiza glabra	Amygdalin+glycyrrhizic acid	In situ self-assembled hydrogel	Hydrogen bonding, hydrophobic association	Co-assembling components	Traumatic brain injury, neural interface	Level 2-3	Mechanical matching to brain tissue not directly measured

Composite-network integration	Tripterygium wilfordii	Celastrol	Metal-coordinated carrier-free hydrogel	Metal-ligand coordination, π–π stacking	Primary gelator and therapeutic component	Cancer immunotherapy	Level 2-3	Narrow therapeutic window, systemic and local toxicity
	Sophora flavescens	Quercetin	Quercetin-ZIF-8/carboxymethyl chitosan injectable hydrogel	Metal–organic framework encapsulation, polymer crosslinking	Therapeutic payload within nanodomain	Diabetic wound healing	Level 2-3	Added complexity over simple drug-loaded hydrogel not justified
	Galla chinensis	Gallic acid	Gallic acid-grafted chitosan/Cu^{2+} hydrogel	Covalent grafting, metal coordination, photothermal crosslinking	Bioactive crosslinker, antioxidant and antibacterial component	Infected wound healing	Level 1-3	Cu^{2+} release kinetics and local toxicity require monitoring
	Epimedium	Icariin	Thiolated icariin-functionalized HA/Col hydrogel	Covalent conjugation, thio-ene crosslinking	Bioactive crosslinker, osteogenic cue	Osteochondral regeneration	Level 2-3	Mechanical strength may be insufficient for load-bearing defects
	Polygonum cuspidatum	Resveratrol	Resveratrol-grafted cellulose nanofiber/PVA–borax	Hydrogen bonding, boronate ester dynamic covalent bonds	Bioactive crosslinker, antioxidant	Wound dressing	Level 1-2	Long-term stability and component release not fully

			hydrogel					characterized
	Coptis chinensis	Berberine	Berberine-HPβCD chitosan thermosensitive hydrogel	Host-guest inclusion, physical gelation	Therapeutic payload	Oral delivery	Level 2	TCM component does not participate in network formation
	Boswellia/Curcuma	Boswellic acid/curcumin	Liposome-in-hydrogel or micelle-in-hydrogel	Nanocarrier encapsulation, polymer network	Therapeutic payload within hierarchical carrier	Cancer, inflammation	Level 2-3	Nanocarrier leakage, manufacturing complexity
	Artemisia argyi	Wormwood essential oil	Carbopol/xanthan gum gel	Physical gelation, viscosity enhancement	Therapeutic payload	Infected diabetic wound	Level 1-2	Extract complexity, quality control
	Hypericum perforatum	Callus extract	Composite hydrogel	Polymer network, extract dispersed	Therapeutic payload	Wound healing, fibrosis prevention	Level 1-2	Batch-to-batch variability of plant extract
Matrix-assisted gelation	TCM formula	Feilin vaginal gel	Carbomer-based gel	Physical gelation	Therapeutic formulation	Cervicitis	Level 2-3	Limited chemical standardization
	TCM formula	Tanreqing gel	Carbomer-based gel	Physical gelation	Therapeutic formulation	Acne	Level 2-3	Marker compounds not fully defined
	Coptis chinensis/Phellodendron	Berberine-containing extract	In situ gel	Ionic crosslinking; physical gelation	Therapeutic payload	Mucosal infection	Level 1-2	In vitro release may overestimate in

vivo retention

Supplementary Table S2 Molecular interactions governing self-assembly of TCM-derived hydrogelators: structural requirements, failure modes, and validation methods.

Molecular interaction	Structural requirements in TCM-associated molecules	Representative molecular classes	Contribution to hydrogel formation	Possible failure modes	Suggested validation methods
π–π stacking	Planar or partially planar aromatic domains with suitable electronic distribution and packing geometry; excessive planarity may favor crystallization	Flavonoids, anthraquinones, phenolic acids, alkaloids, aromatic natural products	Promotes directional molecular packing, nanofiber formation, and long-range supramolecular ordering, reinforces one-dimensional assemblies that entangle into three-dimensional networks.	Strong π–π stacking can cause precipitation or crystallization, weak or sterically hindered interactions may prevent gelation; bulky glycosyl groups disrupt packing.	UV–visible spectroscopy, fluorescence spectroscopy, circular dichroism, X-ray scattering, microscopy, comparison with structurally related analogues lacking aromatic planarity.
Hydrogen bonding	Hydrogen-bond donors and acceptors (hydroxyl, carboxyl, carbonyl, amide, glycosidic, phenolic groups) arranged with suitable spatial accessibility	Saponins, flavonoids, phenolic acids, polyphenols, polysaccharides, glycosides	Stabilizes supramolecular aggregates, supports nanofiber or ribbon formation, strengthens network cohesion, contributes to self-healing or reversible gel-sol transitions.	Hydrogen-bonded networks may weaken in biological fluids because water, proteins, and mucins compete for binding sites, excessive polarity can improve solubility but prevent gelation.	FTIR, variable-temperature spectroscopy, rheology, solvent-competition experiments, gelation tests in biologically relevant media

Hydrophobic association	Hydrophobic domains balanced by hydrophilic groups; amphiphilicity sufficient for aggregation without macroscopic phase separation	Saponins, flavonoids, amphiphilic glycosides, hydrophobic natural products	Drives micelle-like aggregation, vesicular assembly, nanofiber formation, or hydrophobic domain clustering, often a primary force in pH-dependent or amphiphile-driven gelation.	Excessive hydrophobicity causes insoluble precipitation; excessive hydrophilicity prevents aggregation; physiological proteins may bind hydrophobic domains and alter assembly stability.	Critical aggregation concentration, fluorescence probe assays, dynamic light scattering, cryo-electron microscopy, rheology, serum-stability tests.
Electrostatic interactions	Ionizable groups such as protonated amines, carboxylates, phenolates, sulfates, or phosphates with complementary charge distribution	Triterpenoid acids, saponins, monoterpenoid phenols, flavonoids, isoflavones, alkaloids	Regulates ion pairing, co-assembly, morphology transition, network density, and pH-responsive gelation, electrostatic attraction promotes multicomponent assembly	Charge repulsion can prevent aggregation; salt screening can weaken the network, cause unstable aggregation, accelerate gel collapse, or promote burst release	Zeta-potential analysis, pH-dependent gelation studies, salt-resistance tests, isothermal titration calorimetry, rheology, component-release analysis
Metal-ligand coordination	Ligand groups capable of coordinating metal ions, including catechol, gallol, phenolic hydroxyl, carboxylate, or nitrogen-containing groups	Flavonoids, flavonoid glycosides, glycyrrhizic acid, triterpenoid acids, phenolic acids	Provides dynamic crosslinking, network reinforcement, pH responsiveness, adhesion, antioxidant activity, and in some cases antimicrobial function, couples material formation with microenvironmental	Coordination strength depends on pH, oxidation state, metal species, and ligand stoichiometry; excess metal ions may cause cytotoxicity, oxidation or ligand exchange in biological fluids may alter network	UV-visible spectroscopy, X-ray photoelectron spectroscopy, rheology, metal-ion titration, pH-dependent stability tests, cytotoxicity assays, metal-release

			regulation	behavior.	measurements
Host-guest recognition	Complementary host and guest motifs with suitable binding affinity, reversibility, and aqueous compatibility; hosts include cyclodextrins, calixarenes, cucurbiturils, pillararenes	(2-Hydroxypropyl)-β-cyclodextrin, TCM-derived active component–β-cyclodextrin inclusion complexes	Improves solubility of hydrophobic molecules, enables reversible crosslinking, supports injectable supramolecular networks, allows modular incorporation of multiple bioactive components	Binding may be weakened by competing biomolecules, host introduction may shift the system toward a conventional supramolecular polymer hydrogel unless the TCM component has a clearly defined structural role	NMR spectroscopy, isothermal titration calorimetry, competitive binding assays, rheology, release studies, comparison with non-complexed formulations
Van der Waals and cooperative weak interactions	Close molecular packing enabled by complementary shape, conformational flexibility, and appropriate molecular volume; rarely act alone but cooperate with stronger directional interactions	Broadly present across small molecules, glycosides, terpenoids, alkaloids, flavonoids, polysaccharides	Fine-tunes molecular packing; stabilizes assembled domains; contributes to cumulative cohesion of supramolecular networks	Weak and nonspecific, temperature-dependent and difficult to isolate experimentally; over-reliance on this explanation can lead to vague mechanistic interpretation	Molecular simulation, rheology, structure-activity comparison, combined spectroscopy, scattering, and microscopy

Table note. This table expands Table 1 in the main text. It summarizes the principal non-covalent and dynamic interactions that drive TCM-derived hydrogel formation, the structural features required for each interaction, representative molecular classes, characteristic failure modes, and recommended validation methods. The table is intended to guide experimental design and to clarify why apparently similar molecules can differ markedly in gelation behavior.

Supplementary Table S3. Detailed validation requirements for the three engineering routes

Engineering route	Minimun validation	Recommended additional validation	Essential controls	Essential controls
Drug-as-material self-assembly: single-component system	Confirm gel formation, determine critical gelation concentration characterize rheology (storage modulus G′, loss modulus G″), visualize network morphology by TEM/SEM, assess pH and temperature dependence	NMR, ITC, CD, SAXS for molecular packing, time-resolved rheology, stability in serum, mucus, or wound exudate, in vivo retention imaging, pharmacokinetic/biodistribution analysis	Monomer solution at equivalent dose, non-gelling structural analogue or epimer; disrupted assembly control (e.g., by pH shift)	Assuming buffer gelation predicts physiological stability, attributing therapeutic effect to assembled network without comparing with monomer solution, overlooking narrow gelation windows
Drug-as-material self-assembly: multicomponent co-assembly	Demonstrate that co-assembly produces a network not formed by individual components; characterize component ratio in gel, measure component-resolved release; assess rheology and morphology.	NMR, ITC, CD, SAXS to identify intermolecular interactions, comparison with unassembled physical mixture; in vivo retention and biodistribution, combination-index analysis for pharmacological synergy, disruption experiment to test whether assembled structure is required for benefit.	Individual components under identical conditions, physical mixture at same total dose and ratio, assembled hydrogel with disrupted structure, polymer-only or vehicle-only control.	Equating co-delivery with co-assembly, claiming synergy without dose-response or combination-index data, ignoring ionic competition or protein binding in biological fluids.
Composite-network integration: hierarchical carrier-in-carrier	Characterize nanocarrier size, loading, and encapsulation stability, measure release from nanocarrier and from bulk hydrogel, assess rheology and injectability, verify TCM component distribution within	In vivo retention and biodistribution, comparison with simpler hydrogel containing same total dose, local tissue tolerance; systemic exposure, sterilization compatibility	Polymer hydrogel without nanocarrier; nanocarrier alone, free drug at equivalent dose, same hydrogel without TCM component.	Adding hierarchical complexity without demonstrating benefit over simpler formulation, nanocarrier leakage or burst release undermining spatial confinement, overlooking

	matrix			manufacturing and sterilization challenges.
Composite-network integration: matrix-functionalization	Confirm covalent or coordinated integration of TCM component into polymer network, quantify mechanics, swelling, degradation, and adhesion, measure component release, compare with polymer-only hydrogel	Interfacial characterization, in vivo retention; biodistribution, safety assessment including local histology, long-term stability, batch-to-batch reproducibility	Polymer hydrogel without TCM component; free TCM component, physical mixture of polymer and TCM component, same polymer with inactive analogue.	Polymer matrix obscuring TCM contribution, assuming structural role without control experiments, ignoring selective partitioning or degradation of TCM component during storage.
Matrix-assisted gelation of multicomponent formulation	Compare original TCM formulation with hydrogel formulation for residence time, dosing frequency, local tolerability, and therapeutic consistency, chemically fingerprint formulation before and after hydrogelization, track representative marker components	Long-term stability studies, storage stability, scale-up feasibility, sterilization validation, clinical or pharmacodynamic endpoints, batch-to-batch variability analysis.	Original TCM formulation, simple gel vehicle without active extract, formulation with marker compound omitted.	Claiming material innovation without demonstrating improvement over original formulation; improved efficacy due to prolonged contact rather than new material function; quality control challenges with complex extracts.
All routes: physiological relevance	Assess rheological stability, swelling, degradation, release kinetics, protein interactions, ionic strength effects, and structural integrity under biologically relevant	Time-resolved structural analysis, in situ assembly tracking, pharmacokinetic modeling, safety/toxicology assessment in relevant animal models.	Buffer-only controls, protein-free controls, physiological ionic strength and pH controls.	Relying on PBS release data to predict in vivo performance, ignoring protein adsorption and competitive binding, extrapolating from buffer gelation to tissue stability.

	conditions (serum, wound exudate, mucus, synovial fluid)			
All routes: safety and translational risk	For metal-containing systems: measure metal release kinetics, dose-dependent cytotoxicity, local versus systemic exposure, and accumulation. For complex extracts: heavy metal testing, pesticide residue screening, microbial limits, chemical fingerprinting. For all systems: local tissue histology and inflammatory cytokine measurements at administration site	Long-term implantation studies, chronic inflammatory response assessment, fibrous capsule formation, systemic accumulation of degradation products, regulatory positioning analysis.	Vehicle-only control; free metal ion or free extract control, blank hydrogel without active component.	Assuming natural products are inherently safe; ignoring local toxicity despite low systemic exposure, extrapolating short-term rodent safety to long-term human use.

Table note. This table expands the “minimum validation” column of Table 2 in the main text. It specifies the experimental evidence required to substantiate claims about the role of TCM-derived components in hydrogel formation and function, recommended additional validation, essential controls, and common pitfalls. The requirements are organized by engineering route

Supplementary Table S4. Checklist for physiologically relevant characterization of TCM-associated hydrogels.

Parameter	Standard condition	Biologically relevant condition	Suggested method	Why it matters
Rheological stability	PBS, pH 7.4, 25°C	Serum, wound exudate, mucus, synovial fluid, 37°C	Time-sweep rheology, frequency sweep, temperature ramp	Gelation in buffer does not predict network persistence in protein-rich fluids; many hydrogels soften or dissolve within hours of contact with serum
Swelling behavior	PBS, pH 7.4,	Serum, wound exudate, simulated intestinal fluid	Gravimetric swelling ratio at 37°C, equilibrium swelling time	Swelling kinetics affect release rate, mechanical integrity, and tissue conformability, excessive swelling can cause compression of surrounding tissue
Degradation	PBS, pH 7.4	Serum, enzyme-containing media (collagenase, lysozyme, esterase), wound exudate	Mass loss over time, GPC/SEC for molecular weight change, SEM for morphological change	Degradation rate determines residence time and release duration, degradation products may provoke inflammation or systemic effects
Release kinetics	PBS, pH 7.4	Serum, wound exudate, mucus, synovial fluid	HPLC or LC-MS for component-resolved release, cumulative release vs. time, ratio of components released	Release in buffer often overestimates in vivo release, component ratios may change during release, undermining multi-target design
Serum, wound exudate, mucus, synovial fluid	None	Serum albumin, mucin, fibrinogen, lysozyme	ITC, SPR, fluorescence quenching, dynamic light scattering	Proteins can bind to hydrogel components, alter network stability, compete with binding sites, and change release kinetics
Ionic strength effects	NaCl	Physiological ion concentrations (Na^+, K^+, Ca^{2+}, Mg^{2+}, Cl^-)	Rheology at varying ionic strength, DLS; zeta potential, stability tests	Electrostatic assembly is highly sensitive to salt screening, hydrogels that form in low-ionic-strength buffer may collapse in physiological fluids
Structural integrity	Buffer	Biological fluids	Cryo-TEM, SAXS, confocal	Nanoscale architecture may reorganize or

			microscopy, fluorescence correlation spectroscopy	disassemble in biological fluids; structural integrity is often assumed rather than measured
Mechanical matching	Not applicable	Target tissue (e.g., brain, cartilage, skin)	Compressive modulus, tensile modulus, adhesive strength	Mismatch between hydrogel and tissue mechanics can cause secondary injury or implant failure; especially important for neural and orthopedic applications
Sterilization compatibility	Not applicable	After autoclaving, gamma irradiation, or filtration	Rheology, release, and bioactivity before and after sterilization	Sterilization can alter network structure, degrade active components, or change release kinetics; compatibility must be demonstrated, not assumed
Storage stability	Not applicable	After storage at 4°C, 25°C, or 37°C for defined periods	Chemical fingerprinting, rheology, release, and bioactivity at intervals	Shelf stability is a prerequisite for clinical translation; component ratios may change during storage, particularly for complex extracts

Table note. This checklist is intended as a minimum set of parameters for studies that aim to evaluate TCM-derived hydrogels under conditions relevant to their intended application. Not every parameter applies to every system. For example, ionic strength effects are critical for electrostatically assembled hydrogels but may be less important for covalently crosslinked networks. Mechanical matching is essential for neural and orthopedic applications but not for ocular or topical formulations. The checklist is organized to help authors design physiologically relevant characterization and to help readers assess whether a study’s claims are supported by appropriate evidence.
